# Transcriptome analysis reveals insight into molecular hydrogen-induced cadmium tolerance in alfalfa: the prominent role of sulfur and (homo)glutathione metabolism

**DOI:** 10.1186/s12870-020-2272-2

**Published:** 2020-02-04

**Authors:** Weiti Cui, Ping Yao, Jincheng Pan, Chen Dai, Hong Cao, Zhiyu Chen, Shiting Zhang, Sheng Xu, Wenbiao Shen

**Affiliations:** 1 College of Life Sciences, Laboratory Center of Life Sciences, Nanjing Agricultural University, Nanjing, 210095 China; 2 Institute of Botany, Jiangsu Province and Chinese Academy of Sciences, Nanjing, 210014 China; 3 Center of Hydrogen Science, Shanghai Jiao Tong University, Shanghai, 200240 China

**Keywords:** Cadmium, (homo)glutathione, *Medicago sativa*, Molecular hydrogen, RNA-Seq

## Abstract

**Background:**

Hydrogen gas (H_2_) is hypothesised to play a role in plants that are coping with stresses by regulating signal transduction and gene expression. Although the beneficial role of H_2_ in plant tolerance to cadmium (Cd) has been investigated previously, the corresponding mechanism has not been elucidated. In this report, the transcriptomes of alfalfa seedling roots under Cd and/or hydrogen-rich water (HRW) treatment were first analysed. Then, the sulfur metabolism pathways were focused on and further investigated by pharmacological and genetic approaches.

**Results:**

A total of 1968 differentially expressed genes (DEGs) in alfalfa seedling roots under Cd and/or HRW treatment were identified by RNA-Seq. The DEGs were classified into many clusters, including glutathione (GSH) metabolism, oxidative stress, and ATP-binding cassette (ABC) transporters. The results validated by RT-qPCR showed that the levels of relevant genes involved in sulfur metabolism were enhanced by HRW under Cd treatment, especially the genes involved in (homo)glutathione metabolism. Additional experiments carried out with a glutathione synthesis inhibitor and *Arabidopsis thaliana cad2–1* mutant plants suggested the prominent role of glutathione in HRW-induced Cd tolerance. These results were in accordance with the effects of HRW on the contents of (homo)glutathione and (homo)phytochelatins and in alleviating oxidative stress under Cd stress. In addition, the HRW-induced alleviation of Cd toxicity might also be caused by a decrease in available Cd in seedling roots, achieved through ABC transporter-mediated secretion.

**Conclusions:**

Taken together, the results of our study indicate that H_2_ regulated the expression of genes relevant to sulfur and glutathione metabolism and enhanced glutathione metabolism which resulted in Cd tolerance by activating antioxidation and Cd chelation. These results may help to elucidate the mechanism governing H_2_-induced Cd tolerance in alfalfa.

## Background

Hydrogen gas (H_2_) has recently emerged as a molecule that plays physical regulatory roles in plants and animal models. The emission of molecular hydrogen from plants was reported several decades ago [1, 2]. Although the mechanism governing H_2_ production in higher plants remains elusive, the bioregulatory role of H_2_ has been revealed in recent years. Similar to known gasotransmitters, the endogenous functions of H_2_ can be mimicked by the exogenous application of hydrogen-rich water (HRW) (also in animals by hydrogen-rich saline; [3–7]). In plants, H_2_ was found to play roles in root formation by interacting with the nitric oxide and haem oxygenase-1/carbon monoxide pathways [8, 9]. Interestingly, accumulating reports have shown that H_2_ production is elevated in many plant species under abiotic stresses, such as paraquat, NaCl, high light, UV-A, UV-B, and heavy metal stresses, and H_2_ can further act as a regulator in plants coping with abiotic stresses [5, 10–17].

Cadmium (Cd) is a toxic element that seriously threatens crop products and human health [18]. In most plant species, micromolar doses of Cd can cause negative effects, including growth inhibition, water uptake and nutrient metabolism disorders, redox homeostasis imbalance, and even plant death [19, 20]. Generally, one of the prominent disorders caused by Cd toxicity is oxidative stress, which is known as a result of reactive oxygen species (ROS) overproduction. Accumulating evidence has indicated that cellular nonprotein thiols, such as glutathione (GSH), play important roles in ROS scavenging and Cd detoxification [21, 22]. Thus, sulfur and GSH metabolism are important processes in plants coping with Cd [23–26]. Recent reports have indicated that H_2_ could enhance Cd tolerance by increasing antioxidant capacities, including the gene expression of superoxide dismutase (SOD), peroxidase (POD), ascorbate peroxidase (APX), and catalase (CAT), and by reestablishing reduced glutathione homeostasis in *Medicago sativa* and *Brassica campestris*, respectively [15, 27]. However, the underlying mechanism governing glutathione metabolism in H_2_-induced Cd tolerance has not been fully elucidated.

In plants, sulfur metabolism starts from sulfate transport and assimilation. A multigene family of sulfate transporters has been identified in plants, e.g., high affinity sulfate transporters that take in sulfate into root cells were described [28]. Once sulfate is inside the cells, it can be assimilated by ATP sulfurylase and 5′-adenylylsulfate reductase to form sulfite. Then, sulfite reductase, *O*-acetylserine(thiol)lyase, and serine acetyltransferase (SAT) are needed to catalyse the reaction between sulfite and serine to form cysteine [29]. Cysteine is the central compound in the production of methionine and GSH through the activities of cystathionine γ-synthase, cystathionine beta-lyase, homocysteine *S*-methyltransferase, glutamate-cysteine ligase B, and glutathione synthetase [28, 29]. Methionine is the precursor of nicotianamine, which plays a role in ion homeostasis and can be synthesized from the enzymes *S*-adenosylmethionine synthase and nicotianamine synthase [30]. Among the low-molecular-mass thiols, GSH plays an indispensable role in improving oxidative damage, particularly in collaboration with glutathione reductase (GR) and glutathione *S*-transferase (GST). GR is an important antioxidant enzyme that plays a role in the ROS scavenging system, namely, the ascorbate (AsA)-glutathione-nicotinamide adenine dinucleotide phosphate (NADPH) system (also known as the AsA-GSH cycle) [21, 24]. GSTs are ubiquitous proteins encoded by a large gene family that have multiple functions, including cell protection from environmental stress-induced oxidative damage [31]. Meanwhile, the requirements of reduced NADPH in the glutathione reduction process can be produced by NADP-dependent isocitrate dehydrogenase, decarboxylating-like 6-phosphogluconate dehydrogenase, and glucose-6-phosphate 1-dehydrogenase [32]. In legumes, these enzymes can partially produce homoglutathione (hGSH) and homophytochelatins (hPCs) instead of GSH and (PCs) to chelate Cd and act as antioxidants by cysteine sulfhydryl groups [33]. Evidence has shown that the overexpression of bacterial *γ-glutamylcysteine synthetase* in the cytosol of poplar results in higher GSH concentrations, lower superoxide radical (O_2_^.-^) and hydrogen peroxide (H_2_O_2_) concentrations and improved tolerance to Cd [34]. An increase in Indian mustard root glutathione levels, achieved by expressing the bacterial *GR* gene, showed Cd tolerance and accumulation [35]. In addition, the overexpression of *serine acetyltransferase* and *cysteine synthase* can result in an increased level of cysteine and *γ*-glutamylcysteine (*γ*-EC) in tobacco, thereby ultimately leading to the synthesis of PCs to chelate Cd [36]. Research on Massai grass has shown that the synthesis of GSH and PCs is increased under proper S supply, which confers the tolerance of this plant to Cd [37]. Furthermore, after synthesis, (h)PCs can bind Cd to form (h)PC-Cd complexes and then be transported to vacuoles by ATP-binding-cassette (ABC) transporters [20, 38].

The legume plant alfalfa (*M. sativa*) is a widely cultivated forage due to its high protein levels and good palatability, but it is more likely to be planted in marginal lands because of its strong adaptability and limited cultivated land. Cadmium pollution has expanded rapidly in the last hundred years through human activities, such as mining, waste emissions, and fertilizer abuse. Cadmium stress not only affects herbage yield, cell wall structure, and lignification but also poses serious health risks to animals and humans by enrichment through food chains [39]. Previous research has shown the important role of redox status in H_2_-enhanced Cd tolerance and decreased Cd uptake in alfalfa, but little is known about the relative mechanisms involved [27]. In this study, RNA-Seq and RT-qPCR technologies were used to analyse the transcriptomic response of H_2_-regulated pathways under Cd stress in *M. sativa* seedling roots. Furthermore, the importance of (homo)glutathione metabolism in H_2_-induced Cd tolerance was examined using pharmacological and genetic approaches. Our results will have implications for the understanding of the regulatory role of H_2_ on Cd tolerance and reveal the physiological functions of H_2_ in plants.

## Results

### Transcriptome sequencing, assembly, gene expression profiles, and validation analysis

Recent reports have shown that HRW can protect plants against Cd stress [15, 27]. To further explore the role of H_2_ in the plant response to Cd stress, endogenous H_2_ levels were detected under Cd stress with or without HRW pretreatment. As detected by gas chromatography, endogenous H_2_ in alfalfa seedling roots was increased by 84.09% after 12 h of Cd stress, and a higher H_2_ content was found after the administration of HRW followed by Cd exposure (Additional file 1: Figure S1). These results, as well as those of previous reports [27], indicated that Cd-stimulated H_2_ production might act as a signal to regulate Cd resistance. However, the underlying mechanism governing H_2_ signalling and Cd tolerance has not been thoroughly elucidated.

To address this gap, a mixed RNA sample from three independent experiments in alfalfa seedling roots was prepared for RNA-Seq using an Illumina HiSeq™ 2500. We sequenced four groups of complementary DNA (cDNA) libraries, Sample 1 (12 h in 1/4 Hoagland’s solution then changed to fresh 1/4 Hoagland’s solution for another 12 h, Con → Con); Sample 2 (12 h in 1/4 Hoagland’s solution then changed to 12 h of Cd treatment in 1/4 Hoagland’s solution, Con → Cd); Sample 3 (with 12 h of HRW pretreatment followed by 12 h of Cd treatment, HRW → Cd); and Sample 4 (with 12 h of HRW pretreatment then changed to 1/4 Hoagland’s solution for another 12 h, HRW → Con), and generated 60,723,124 sequence reads encompassing 9.24 Gb of sequence data (Additional file 2: Table S1). The sequence reads were aligned to the *Medicago truncatula* reference genome (JCVI Medtr v4) by Tophat software with a setting that allowed two base mismatches. Of the total reads, 54.09% matched multiple (5.46%) or unique (48.6%) genomic locations (Additional file 2: Table S1).

**Fig. 1:**
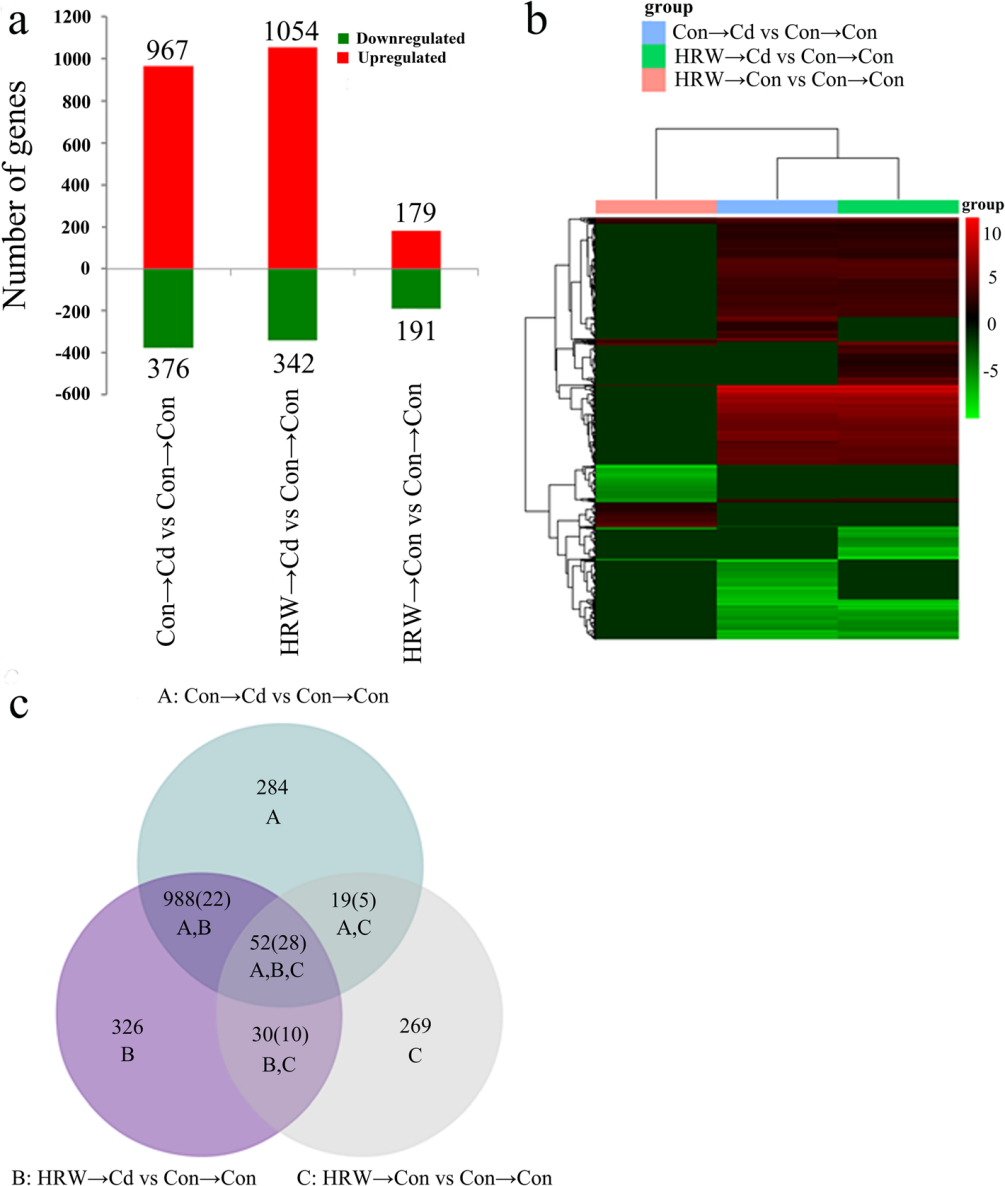
Differential abundance of transcripts involved in H_2_-mediated cadmium (Cd) resistance. Transcriptome of alfalfa seedling roots treated with 100 μM Cd for 12 h, with or without hydrogen-rich water (HRW) pretreatment for 12 h. **a** Summary of significantly upregulated and downregulated transcripts in the three experimental groups. **b** Heatmap showing the increasing or decreasing expression of all the differentially abundant transcripts identified in the three groups. **c** Venn diagram of the differential abundance of transcripts identified in three groups, and the number in brackets indicates the counts of transcripts that exhibited opposite regulatory tendencies

The normalized expression values (fragments per kilobase of transcript, per million fragments mapped, FPKM) of each unigene in alfalfa seedling root libraries with |log2(fold change)| ≥ 3 and *P* value ≤0.05 were considered to be differentially expressed genes (DEGs). We obtained 1968 DEGs, with 1343 being detected between the S2/S1 (Con → Cd vs Con → Con) libraries, 1396 between the S3/S1 (HRW → Cd vs Con → Con) libraries, and 370 between the S4/S1 (HRW → Con vs Con → Con) libraries (Additional file 3: Table S2, Additional file 4: Table S3 and Additional file 5: Table S4). Among the three experimental groups, 967 DEGs were upregulated, and 376 were downregulated, with Cd treatment (S2/S1), while 1054 and 179 DEGs were upregulated, and 342 and 191 DEGs were downregulated, with Cd treatment after HRW pretreatment (S3/S1) and HRW treatment (S4/S1), respectively (Fig. 1a). In addition, a heatmap analysis was performed, clearly showing the notable change in expression ratio between H_2_ and Cd treatment and the control (Fig. 1b). Among the three groups, the common signature of HRW and/or Cd treatment contained 52 transcripts, including 28 DEGs exhibiting opposite regulatory tendencies (Fig. 1c). There were 284, 326, and 269 unique DEGs in the S2/S1, S3/S1, and S4/S1 comparisons, respectively. Meanwhile, 988 DEGs were shared only between Cd treatment alone (S2/S1) and HRW plus Cd (S3/S1) treatment, including 22 DEGs displaying opposite regulatory tendencies (Fig. 1c).

Furthermore, we used RT-qPCR to validate the differences in gene expression determined by the RNA-Seq data. For example, the transcript levels of the *Mtr_7g085630* homologue detected by RT-qPCR in the Con → Cd, HRW → Cd, and HRW → Con groups were upregulated 3.42-, 4.47-, and 1.09-fold compared to the Con → Con group, respectively (Additional file 6: Figure S2); the corresponding transcript levels in the RNA-Seq data were increased 3.77- and 4.13-fold in the two groups (Con → Cd vs Con → Con and HRW → Cd vs Con → Con, respectively) with no data for the HRW → Cd vs Con → Con comparison, which means that the fold change was less than 3 (Additional file 3: Table S2, Additional file 4: Table S3 and Additional file 5: Table S4). Similarly, the transcript levels of the other 8 detected genes showed largely similar trends to the RNA-Seq results, suggesting that the quality of our RNA sequencing data was acceptable.

### Functional annotation and classification

**Fig. 2:**
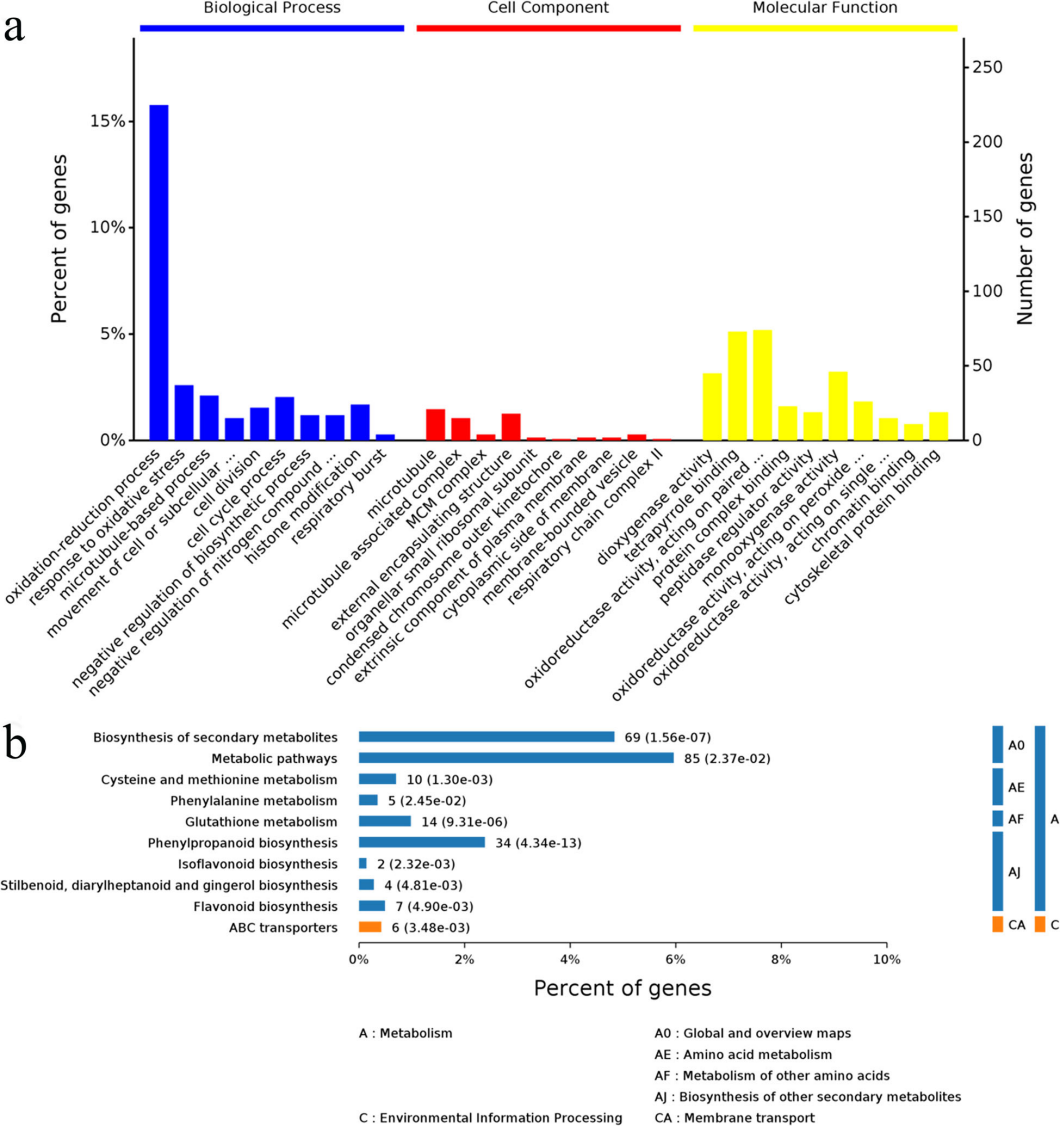
Clusters of unigenes in the *M. sativa* transcriptome. **a** Gene Ontology (GO) terms. The 10 most significantly enriched terms in the level 4 Gene Ontology hierarchy. Information on the percentage and number of involved proteins in a term are shown on the left and right y-axes. **b** Clusters of enriched Kyoto Encyclopedia of Genes and Genomes (KEGG) pathways that are arranged into the metabolism subcategories, and the number of involved proteins in a specific pathway and corresponding *P* value are shown on the right side of column

Gene Ontology (GO) classification was performed to functionally categorize the significantly changed genes into three main categories: biological process (BP), cellular function (CC), and molecular function (MF). In our results, 1221 genes were annotated with BP, and 242 terms were significantly enriched. In contrast, 532 genes were annotated with CC, 30 terms were significantly enriched, and 1234 genes were annotated with MF, including 106 terms that were significantly enriched (Additional file 7: Figure S3). Moreover, the top 10 significantly enriched terms by GO hierarchy (in level 4) are depicted (Fig. 2a). Among the groups, 48 BPs were significantly enriched (*P* value≤0.05) at level 4, including the oxidation-reduction process (GO:0055114), the regulation of cellular metabolic process (GO:0031323), the cellular amino acid metabolic process (GO:0006520), the response to oxidative stress (GO:0006979), the microtubule-based process (GO:0007017), the cell cycle process (GO:0022402), the cell division (GO:0051301), the histone modification process (GO:0016570) (Additional file 8: Table S5), etc.

We further categorized the biological functions of the DEGs by using Kyoto Encyclopedia of Genes and Genomes (KEGG) pathway enrichment analysis. The results showed that the biosynthesis of secondary metabolites, cysteine and methionine metabolism, glutathione metabolism, phenylpropanoid biosynthesis, and ABC transporters were significantly enriched metabolic pathways in the H_2_ and/or Cd treatment response (Fig. 2b, Additional file 9: Table S6). From the BP results and KEGG analysis, we noticed that the cysteine and glutathione metabolism pathways identified in the KEGG analysis were well correlated with the oxidation-reduction process and cellular amino acid metabolic process identified in the BP analysis.

### Regulation of sulfur and glutathione metabolism pathways by H_2_ and/or Cd

**Fig. 3:**
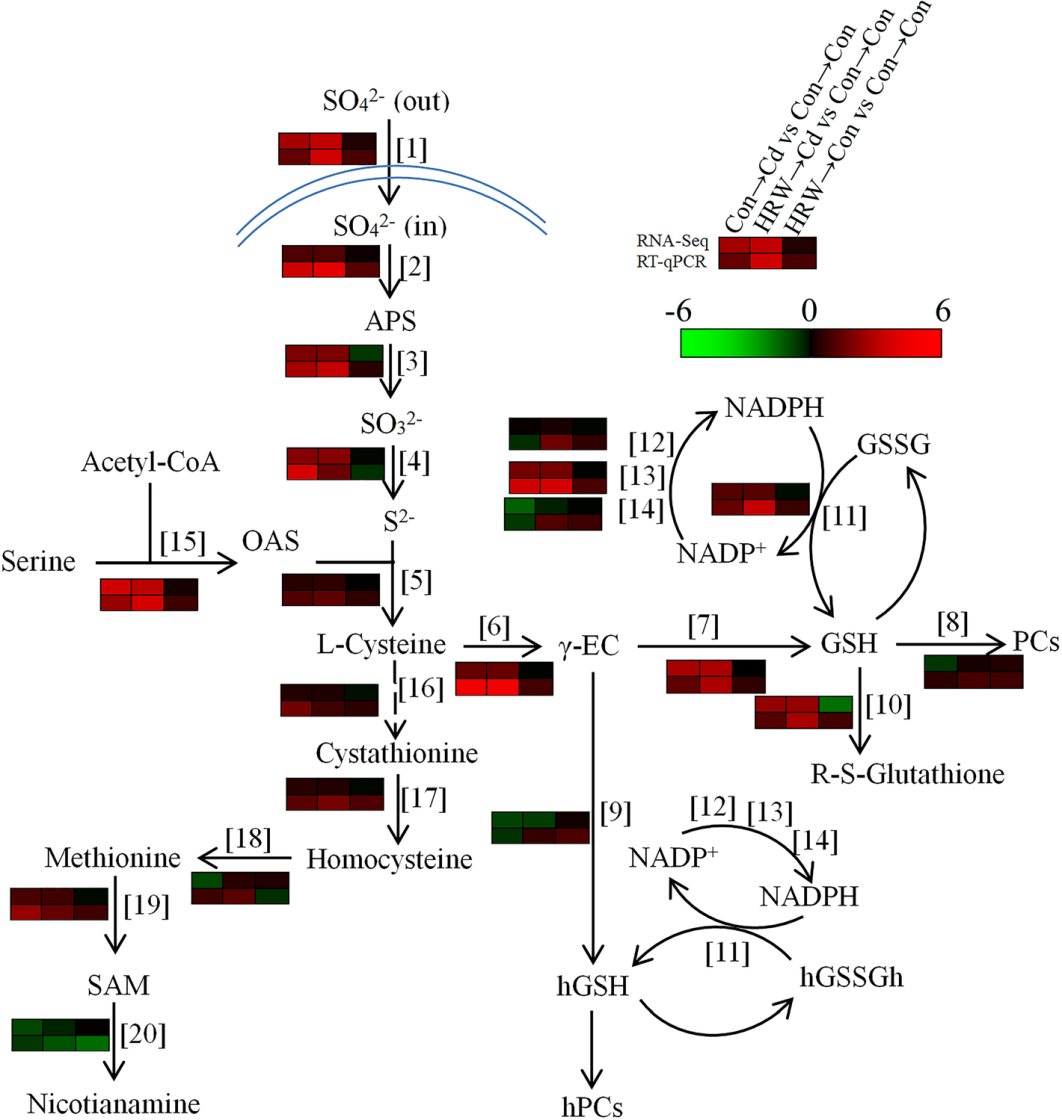
Transcriptional changes in genes identified by RNA-Seq and RT-qPCR involved in sulfur and glutathione metabolism in alfalfa seedling roots. Arrows represent enzymatic reactions. Coloured bars near the arrows indicate the relative expression of the corresponding homologous genes in different treatments. Transcripts detected by RT-qPCR are presented relative to the control samples (Con → Con), with the expression being normalized to two internal reference genes in each sample. Abbreviations: APS, adenosine-phosphosulfate; *γ*-EC, *γ*-glutamylcysteine; GSH, reduced glutathione; GSSG, oxidized glutathione; PCs, phytochelatins; hGSH, reduced homoglutathione; hGSSGh, oxidized homoglutathione; hPCs, homophytochelatins; OAS, *O*-acetyl serine; SAM, *S*-adenosyl-methionine. [1], high affinity sulfate transporter type 1 (Mtr_3g073780 homologue); [2], ATP sulfurylase (Mtr_1g102550 homologue); [3], 5′-adenylylsulfate reductase (Mtr_2g023540 homologue); [4], sulfite reductase [ferredoxin] protein (Mtr_4g077190 homologue); [5], *O*-acetylserine(thiol)lyase (Mtr_5g006340 homologue); [6], glutamate-cysteine ligase B (Mtr_8g098350 homologue); [7], glutathione synthetase (Mtr_7g113890 homologue); [8], phytochelatin synthase (Mtr_7g097190 homologue); [9], homoglutathione synthetase (Mtr_7g113880 homologue); [10], glutathione S-transferase (Mtr_2g070070 homologue); [11], glutathione reductase (Mtr_6g033515 homologue); [12], NADP-dependent isocitrate dehydrogenase (Mtr_2g062840 homologue); [13], decarboxylating-like 6-phosphogluconate dehydrogenase (Mtr_7g017900 homologue); [14], glucose-6-phosphate 1-dehydrogenase (Mtr_7g111760 homologue); [15],serine acetyltransferase (Mtr_3g058410 homologue); [16], cystathionine γ-synthase (Mtr_7g011230 homologue); [17], cystathionine beta-lyase (Mtr_1g064320 homologue); [18], homocysteine *S*-methyltransferase (Mtr_1g103290 homologue); [19], *S*-adenosylmethionine synthase (Mtr_2g046710 homologue); [20], nicotianamine synthase (Mtr_1g084050 homologue)

It is well known that glutathione plays an important role in plant resistance to Cd by alleviating oxidative stress and chelating Cd with PCs [21, 22]. To further investigate the roles of sulfur assimilation, cysteine and methionine metabolism, and glutathione metabolism in H_2_-induced Cd resistance, we examined the transcriptional regulation of sulfur and glutathione metabolism in H_2_ and/or Cd treatment from the RNA-Seq data and further compared the results by RT-qPCR (Fig. 3).

Two sulfate transporter genes were identified as significantly expressed in alfalfa seedling roots, and both were upregulated by Cd stress with or without HRW pretreatment (Fig. 3, Additional file 10: Table S7). ATP sulfurylases and 5′-adenylylsulfate reductases participate in sulfate activation followed by its reduction to sulfide. In this study, three identified ATP sulfurylases and one 5′-adenylylsulfate reductase were upregulated by HRW plus Cd treatment. Sulfite can be reduced to sulfide by sulfite reductase, which was upregulated under Cd stress and showed an increasing trend after HRW pretreatment in the RNA-Seq data. Sulfide is incorporated with *O*-acetylserine by *O*-acetylserine(thiol)lyase to form cysteine, and their transcripts were distinctly upregulated by HRW pretreatment in the RT-qPCR experiment. In alfalfa plants, glutamate-cysteine ligase (GCL; also known as *γ*-glutamylcysteine synthetase, *γ*-ECS) catalyses the first step in glutathione (GSH) and homoglutathione (hGSH) synthesis followed by incorporation with glycine to form GSH by glutathione synthetase (GS) and combination with alanine to form hGSH by homoglutathione synthetase (hGS [40];). With RNA-Seq and confirmation by RT-qPCR, we observed that the transcripts of *GCL* and *GS* were both upregulated in the Cd plus HRW treatment. We also noticed that the transcripts of *hGS* were decreased by Cd, but this reduction was determined to alleviated both in RNA-Seq and RT-qPCR data in Cd plus HRW treatment (by 8.3 and 42.5% compared to Cd alone treatment, respectively). The transcripts of *glutathione S-transferase* (*GST*) were increased by HRW pretreatment under Cd stress. Meanwhile, the expression of *phytochelatin synthase* (*PCS*) and *homoglutathione synthetase* genes was downregulated by Cd, but recovered when HRW was applied. Glutathione reductase (GR) catalyses the change in the oxidative form of glutathione to a reduced form with reduced nicotinamide adenine dinucleotide phosphate (NADPH) as the reducing power [35]. In this study, transcripts of *GR* and *6-phosphogluconate dehydrogenase* were increased under Cd and HRW plus Cd treatment, but the transcripts of *NADP-dependent isocitrate dehydrogenase* and *glucose-6-phosphate 1-dehydrogenase* were decreased by Cd and further reversed by HRW pretreatment. Methionine can be catalysed from cysteine by cystathionine beta-lyase and homocysteine *S*-methyltransferase, and their expression was slightly increased by HRW under Cd stress. Moreover, methionine is the precursor of nicotianamine, and the expression of *nicotianamine synthase* was decreased by Cd treatment and further downregulated in samples treated with HRW plus Cd (Fig. 3, Additional file 10: Table S7).

Furthermore, we measured the sulfur contents in the medium before and after HRW and Cd treatment. The results showed that sulfur contents were decreased by 9.8 and 7.6% after 12 h of pretreatment with or without HRW, respectively (Additional file 11: Figure S4a). Next, Hoagland’s solution was changed and decreased to 92.9 and 58.5% after 12 h and 5 d of alfalfa seedling growth, respectively. Interestingly, Cd stress alone induced a 3.8% sulfur reduction after 12 h of treatment and a 25.4% decrease after 5 d of treatment compared to a 5.4 and 47.8% loss, respectively, when there was HRW pretreatment (Additional file 11: Figure S4a). Meanwhile, we determined the sulfur content in alfalfa seedlings after 12 h and 5 d of Cd treatment. The total sulfur in the samples was slightly but not significantly increased after 12 h of Cd treatment. However, there were significant differences in protein-bound sulfur samples. For example, HRW pretreatment led to an increase in protein-bound sulfur by 18.1 and 22.5% with Cd stress and 10.8 and 13.0% without Cd stress aboveground and underground, respectively (Additional file 11: Figure S4b-e). When stressed with 100 μM Cd for 5 d, total sulfur contents decreased notably (aboveground: by 16.7%, underground: by 20.8%), but this effect was alleviated with HRW pretreatment, particularly underground where sulfur contents were reduced by only 7.2% compared to Con). Similarly, protein-bound sulfur contents were also decreased after 5 d of Cd stress and were mitigated by HRW pretreatment (increased by 6.7% in aboveground parts and 16.0% in underground parts in samples with HRW pretreatment compared to Cd-stress-alone samples) (Additional file 11: Figure S4b-e).

### H_2_-induced Cd tolerance was closely related to the amount of available cellular (homo)glutathione

**Fig. 4:**
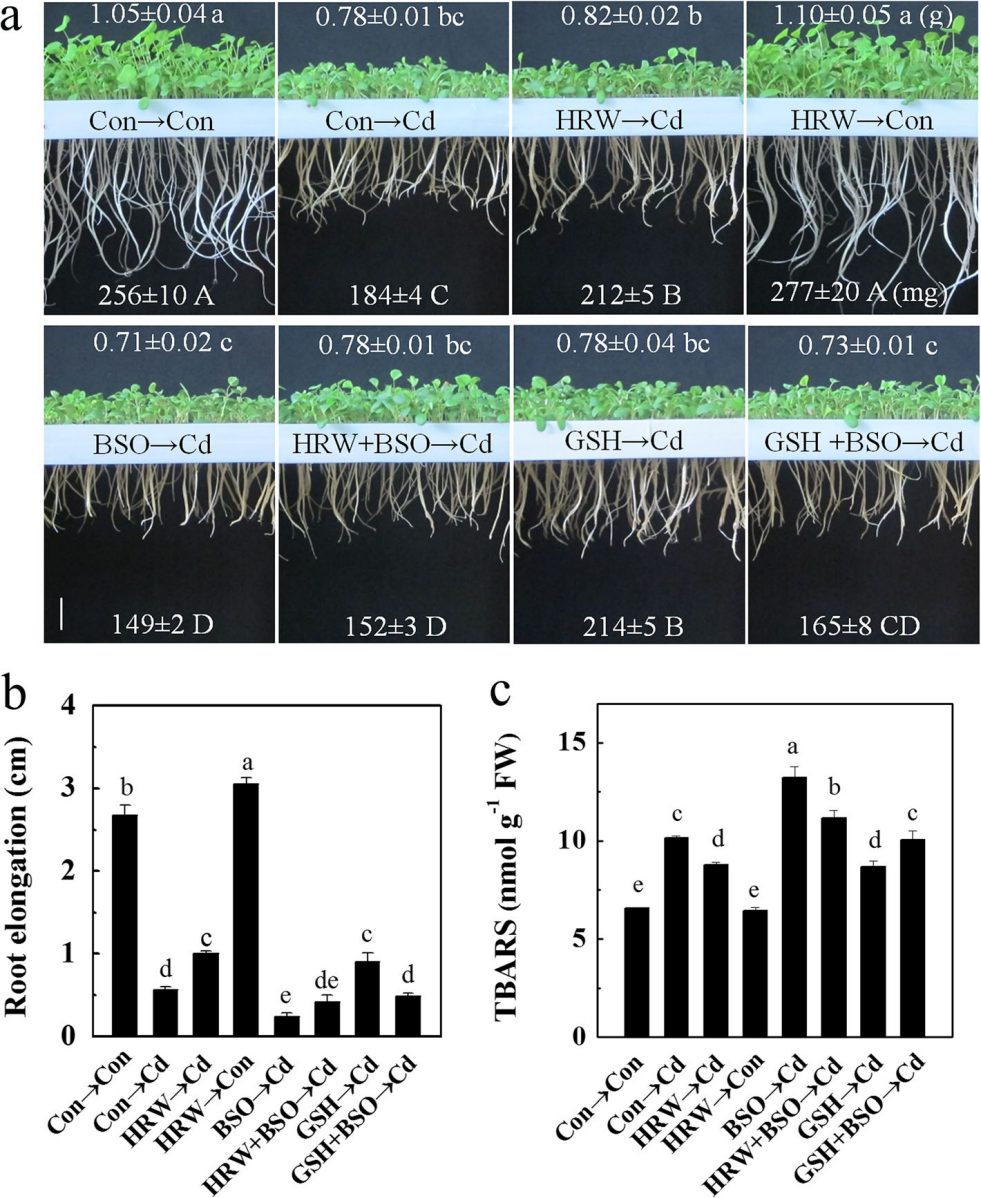
Effects of H_2_, _L_-buthionine-*S*,*R*-sulfoximine (BSO), and reduced glutathione (GSH) on the Cd-induced inhibition of alfalfa seedling growth and fresh weight (**a**), root elongation (**b**), and thiobarbituric acid-reactive substance (TBARS) content (**c**) in alfalfa roots. Five-day-old seedlings were pretreated with or without HRW, 500 μM BSO, and 1 mM GSH individually or in combination for 12 h followed by another 72 h (**a** and **b**) and 24 h (**c**) treatment with 100 μM CdCl_2_. Data in the top and bottom of part (**a**) indicate the fresh weight of 30 seedlings above ground and underground, respectively. Bar = 2 cm. Values are the mean ± SE of three independent experiments with at least three replicates for each. Bars with different letters indicate significant differences (*P* < 0.05) according to Duncan’s multiple range test

**Fig. 5:**
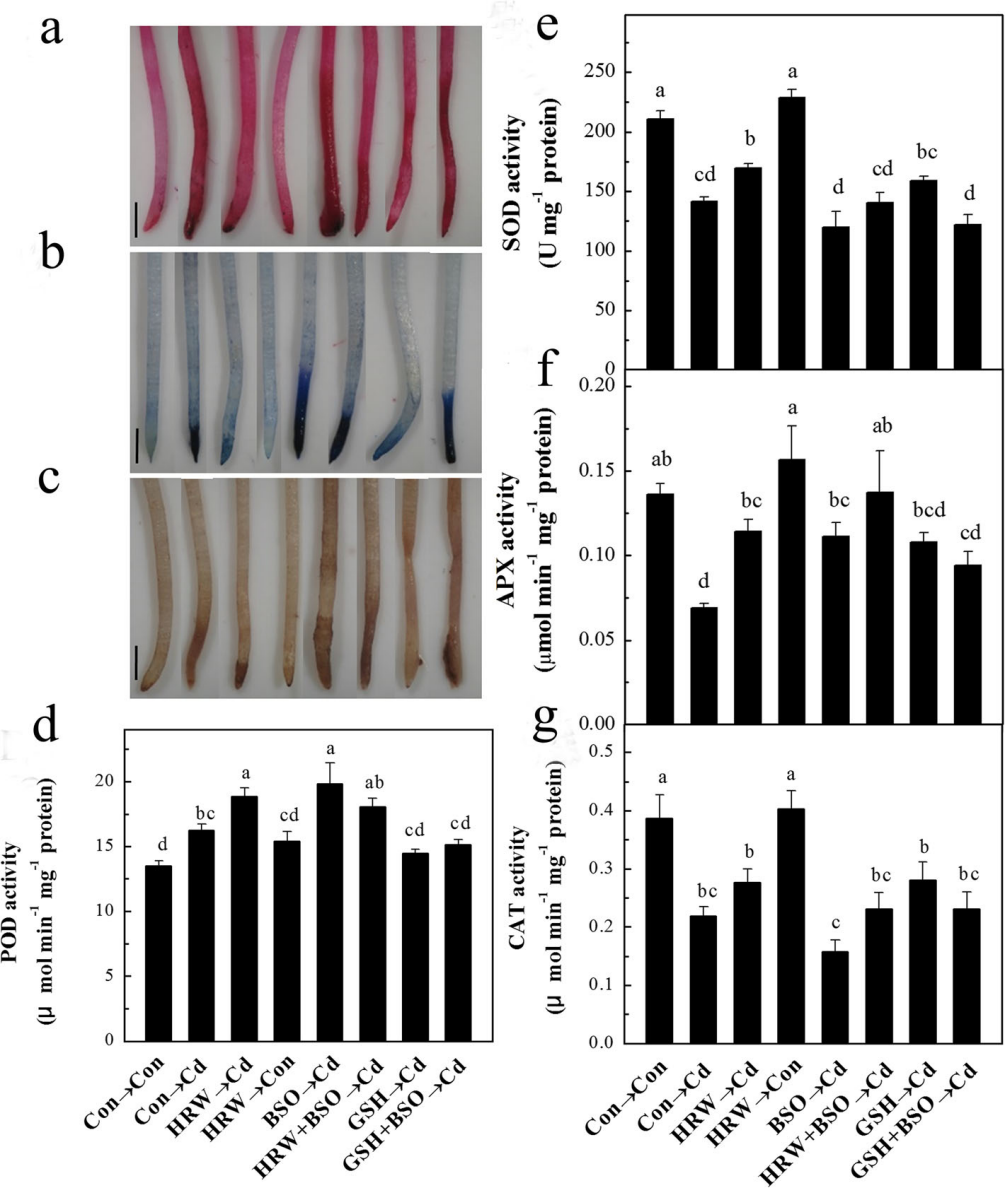
Effects of H_2_, BSO, GSH, and Cd on lipid peroxidation (**a**), loss of plasma membrane integrity (**b**), localization of reactive oxygen species (ROS) (**c**), and the activity of peroxidase (POD) (**d**), superoxide dismutase (SOD) (**e**), ascorbate peroxidase (APX) (**f**), and catalase (CAT) (**g**) in alfalfa seedling roots. Five-day-old seedlings were pretreated with or without HRW, 500 μM BSO, and 1 mM GSH individually or in combination for 12 h followed by another 24 h treatment with 100 μM CdCl_2_. Bar = 1 mm. Values are the mean ± SE of three independent experiments with at least three replicates for each. Bars with different letters indicate significant differences (*P* < 0.05) according to Duncan’s multiple range test

Among the biothiols, glutathione is recognized as the heart of the redox hub [21]. To demonstrate the important role of glutathione in H_2_-induced Cd resistance, a pharmacological test was performed by using _L_-buthionine-*S*,*R*-sulfoximine (BSO), a specific inhibitor of *γ*-glutamylcysteine synthesis (*γ*-ECS), which was successfully used to block endogenous glutathione synthesis in plants [41, 42]. Compared with samples treated with Cd alone, Cd-stressed samples with BSO pretreatment showed more serious growth inhibition (Fig. 4a). Furthermore, the seedling growth inhibition alleviated by HRW was reversed by BSO (Fig. 4a, b). Moreover, the addition of GSH together with BSO pretreatment for 12 h resulted in a slight alleviation of alfalfa seedling growth inhibition under another 72 h of Cd stress (Fig. 4a, b). This result suggested the important role of the endogenous production of glutathione in the 3-d period of Cd stress in plants. In addition, lipid peroxidation in seedling roots was detected by measuring the content of thiobarbituric acid-reactive substance (TBARS). The results showed a sharp increase in TBARS content due to BSO pretreatment under Cd stress, and this increase was reversed by the addition of GSH. BSO pretreatment also inverted the protective role of H_2_ in Cd-induced lipid peroxidation (Fig. 4c). Moreover, the lipid peroxidation, loss of plasma membrane integrity, and ROS accumulation detected by histochemical staining in alfalfa seedling roots showed similar results (Fig. 5a-c).

Similarly, the genetic results from the *Arabidopsis thaliana cad2–1* mutant showed that the inhibition of seedling root growth was more serious in mutant plants, and the protective role of HRW in Cd stress was blocked in *cad2–1* plants (Additional file 12: Figure S5a, b). By using the fluorescent dye monochlorobimane, GSH content in WT plant roots was detected, and the result showed an increase after Cd treatment. Furthermore, this effect was enhanced by either HRW or exogenous GSH treatment. In contrast, there was only slight fluorescence in *cad2–1* plant roots under Cd and/or HRW treatment, but the signal became brighter after exogenous GSH pretreatment (Additional file 12: Figure S5c). Interestingly, the Cd concentration was increased in *cad2–1* seedling roots, which could be mitigated by HRW or GSH (Additional file 12: Figure S5d). In addition, the plasma membrane integrity loss and lipid peroxidation induced by Cd treatment were aggravated in *cad2–1* seedling roots, and these characteristics were insensitive to HRW but not GSH in *cad2–1* mutant plants compared to WT plants (Additional file 12: Figure S5e, f).

Furthermore, we investigated the activities of several important antioxidant enzymes in alfalfa seedling roots. The results showed that Cd treatment caused a 20.12% increase in POD activity, but the increases reached 39.62 and 46.55% after HRW or BSO pretreatment, respectively (Fig. 5d). We noticed that compared with HRW or BSO pretreatment alone, pretreatment with HRW and BSO together slightly decreased POD activity. Furthermore, the results revealed a significant decrease in SOD, APX, and CAT activities in alfalfa seedling roots under Cd stress (Fig. 5e-g). SOD and CAT activities were slightly decreased with BSO pretreatment compared to Cd treatment alone, but APX activity was increased by BSO pretreatment. Moreover, compared to pretreatment with BSO alone, the BSO plus HRW pretreatment exhibited slight but not significant increases in SOD, APX, and CAT activities. Furthermore, the samples pretreated with GSH plus BSO showed significantly alleviated POD activity compared with those pretreated with BSO alone, but GSH showed little effect on SOD, APX, and CAT activities under BSO plus Cd treatment (Fig. 5d-g).

**Fig. 6:**
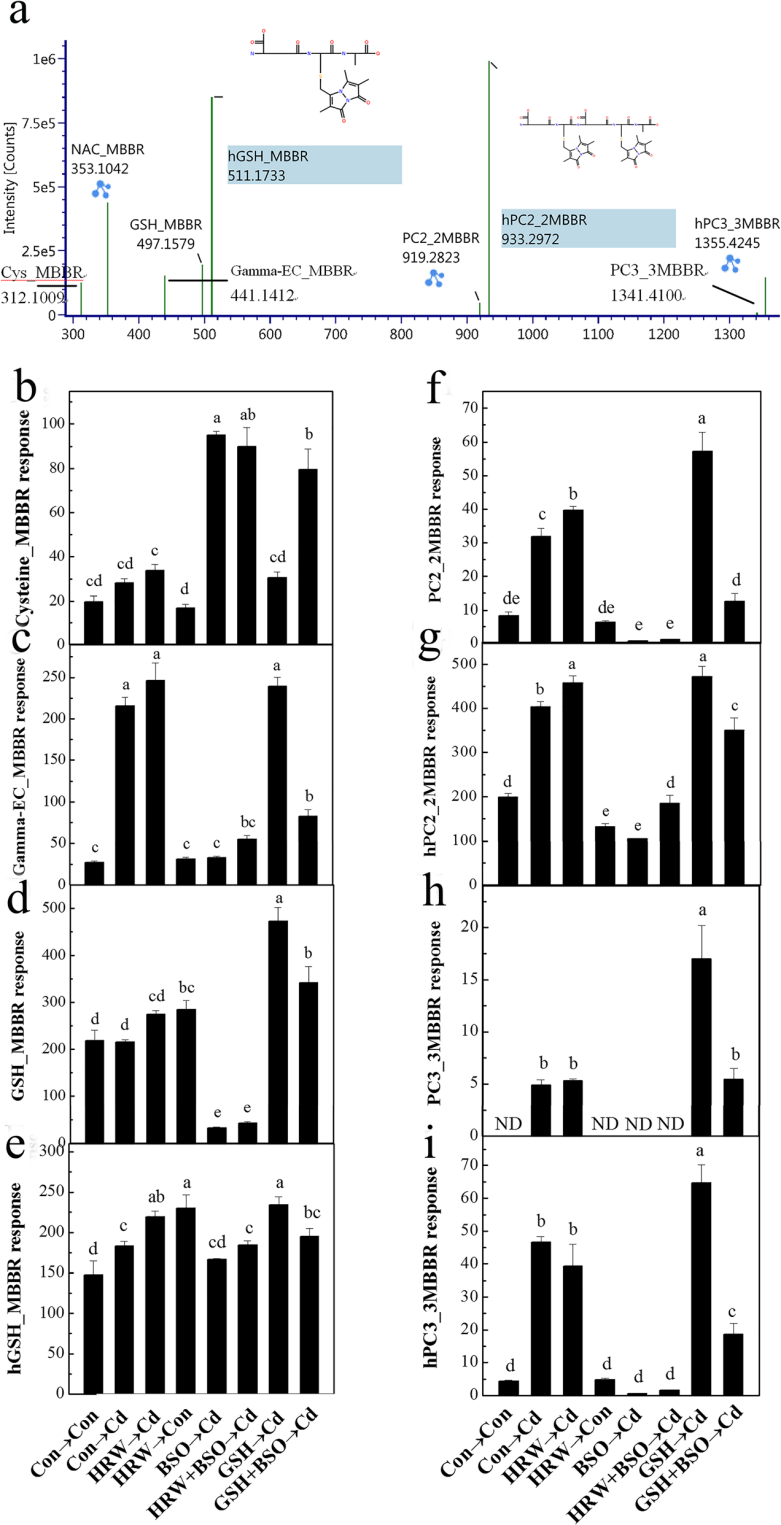
Effects of H_2_, BSO, GSH, and Cd on the mass spectra of cysteine (**b**), *γ*-glutamylcysteine (*γ*-EC) (**c**), GSH (**d**), homoglutathione (hGSH) (**e**), PC2 (**f**), hPC2 (**g**), PC3 (**h**), and hPC3 (**i**) with MBBR in alfalfa seedling roots. Five-day-old seedlings were pretreated with or without HRW, 500 μM BSO, and 1 mM GSH individually or in combination for 12 h followed by another 12-h treatment with 100 μM CdCl_2_. **a** Mass spectra of biothiols in the *m/z* ranges. ND, not detected. Values are the mean ± SE of three independent experiments with two replicates for each. Bars with different letters indicate significant differences (*P* < 0.05) according to Duncan’s multiple range test

Additional experiments were carried out to detect the amounts of PCs in alfalfa seedling roots by ultra-performance liquid chromatography-electrospray ionization-quadrupole time-of-flight tandem mass spectrometry (UPLC-ESI-QTOF/MS). After derivatization with monobromobimane (MBBR), the relative contents of PCs and their precursors under HRW/Cd treatment were determined (by using *N*-acetylcysteine as the internal standard). As shown in Fig. 6, the contents of *γ*-EC, hGSH, PC_2_, hPC_2_, PC_3_, and hPC_3_ were increased under Cd stress, which suggested a positive response in alfalfa seedlings. Compared to Cd treatment alone, HRW pretreatment increased the contents of cysteine, *γ*-EC, GSH, hGSH, PC_2_, and hPC_2_ (especially hGSH, PC_2_ and hPC_2_) (Fig. 6b-g). Interestingly, the results of HRW treatment alone showed a significant increase in GSH and hGSH contents (Fig. 6d, e). The concentration of GSH was significantly decreased after the addition of BSO; meanwhile, the cysteine level was sharply increased (Fig. 6b, d). In addition, the exogenous addition of GSH increased the contents of GSH, hGSH, PC_2_, and hPC_2_ under Cd or BSO plus Cd treatment conditions (Fig. 6c-f).

### H_2_ decreased Cd concentration and modulated the transcripts of ABC transporter genes in alfalfa seedling roots

**Fig. 7:**
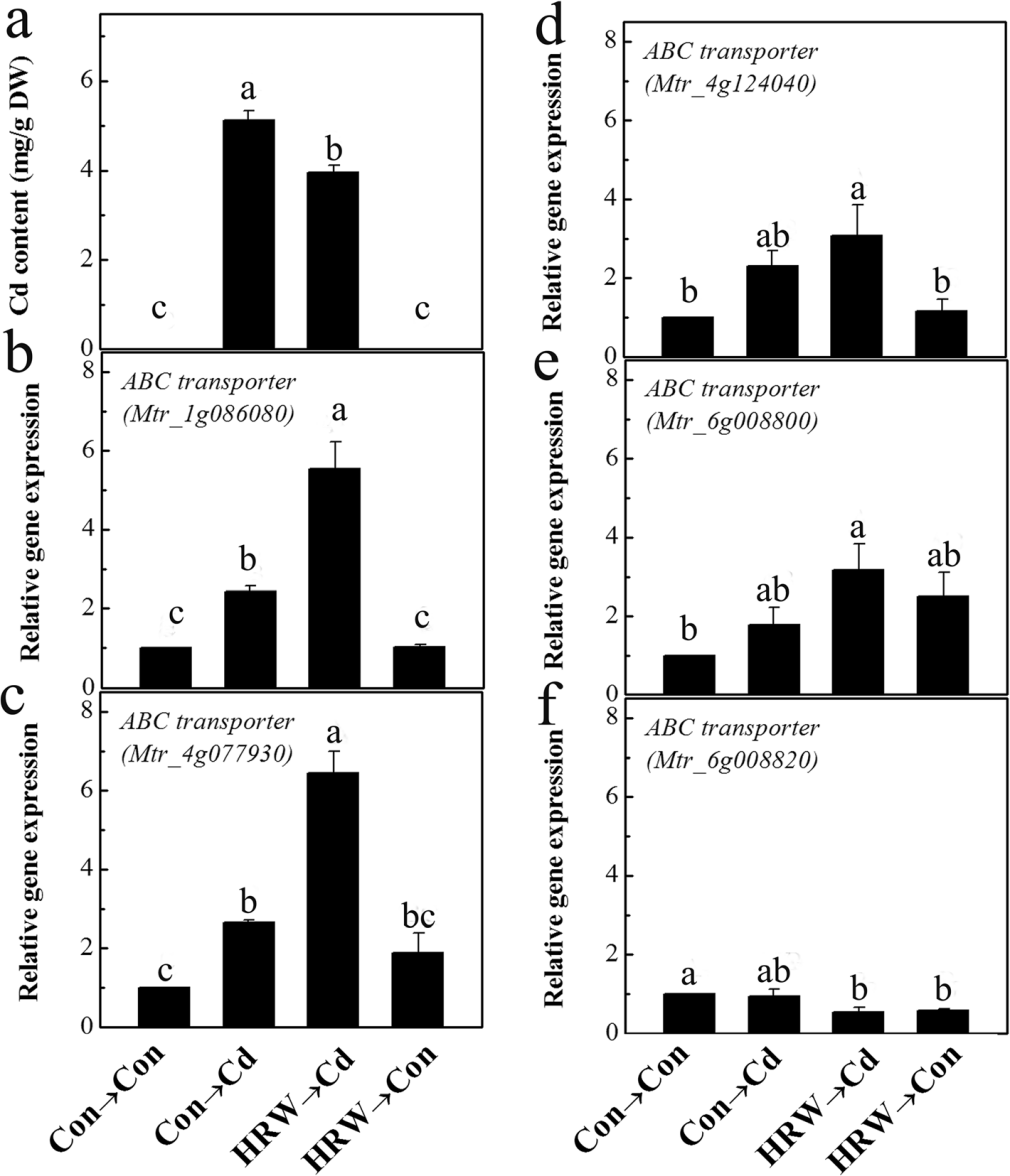
Content of Cd and the transcripts of ABC transporter genes in alfalfa seedling roots. Five-day-old seedlings were pretreated with or without HRW for 12 h followed by another 12 h (**b**-**f**) or 72 h (**a**) treatment with 100 μM CdCl_2_. The Cd content in alfalfa seedling roots was determined after 72 h of Cd exposure (**a**). Relative expression of the *ABC transporter* gene homologues of *Mtr_1g086080* (**b**), *Mtr_4g077930* (**c**), *Mtr_4g124040* (**d**), *Mtr_6g008800* (**e**), and *Mtr_6g008820* (**f**) was detected by RT-qPCR. The sample without chemicals was the control (Con). Expression levels of corresponding genes are presented relative to the control samples and were normalized to the expression of two internal reference genes in each sample. Values are the mean ± SE of three independent experiments with at least three replicates for each. Bars with different letters indicate significant differences (*P* < 0.05) according to Duncan’s multiple range test

The toxicity of Cd in alfalfa seedling roots was estimated by detecting the concentration of Cd and the expression of ABC transporter genes. As shown in Fig. 7a, the Cd concentration in seedling roots was decreased by 23.0% after HRW pretreatment. This result suggested that HRW pretreatment inhibited the uptake of Cd into alfalfa seedling roots. Furthermore, the expression of ABC transporter genes was detected by RT-qPCR. The results showed that the ABC transporter genes *Mtr_1g086080 homologue*, *Mtr_4g077930 homologue*, *Mtr_4g124040 homologue*, and *Mtr_6g008800 homologue* were upregulated by Cd and further intensified by HRW plus Cd treatment (*Mtr_1g086080 homologue* and *Mtr_4g077930 homologue* in particular) (Fig. 7b-e). Meanwhile, we found that the transcript of the *Mtr_6g008820 homologue* was decreased by HRW pretreatment under Cd or Con conditions (Fig. 7f).

## Discussion

Molecular hydrogen (H_2_) is suggested to be a bioregulator in plant defence against environmental stresses [6, 43]. Previous work has documented the role of H_2_ in plants coping with Cd. For example, Wu et al. [15] found that Cd stress increased molecular hydrogen accumulation in *Brassica campestris*, which is similar to its effect on alfalfa (Additional file 1: Figure S1); endogenous H_2_ subsequently reestablished reduced glutathione homeostasis and led to Cd tolerance. In our previous study, it was found that H_2_ can protect alfalfa seedlings from Cd toxicity by regulating redox homeostasis [27]. The underlying mechanisms were identified by iTRAQ, which showed that H_2_-regulated proteins were classified into seven main categories, including sulfur compound metabolic process, oxidation-reduction process, and metal ion homeostasis [44]. However, there are still gaps in the body of knowledge concerning H_2_ activation and Cd tolerance. In this study, we determined H_2_-regulated genes followed by experiments to examine how H_2_ mediated alfalfa resistance to Cd. These results suggest that sulfur and glutathione metabolism might be the key downstream mediators of the H_2_-regulated pathway in Cd-induced signalling.

### Biological processes revealed by RNA-Seq showed the central role of sulfur and glutathione metabolism in H_2_-mediated alfalfa response to Cd stress

In recent years, transcriptome analysis has been successfully used to reveal gene expression profiles and to provide a molecular understanding of biological processes. In this study, transcriptome sequencing analysis helped us to understand the mechanisms underlying H_2_-related signals in the Cd stress response (Additional file 2: Table S1). Among the 1968 identified DEGs (Additional file 3: Table S2, Additional file 4: Table S3 and Additional file 5: Table S4), an increase in the number of DEGs was found after HRW treatment under Cd stress (Fig. 1). In this study, the GO categories and KEGG enrichment analysis revealed the pathways enriched with HRW and/or Cd treatments, such as the oxidation-reduction process, regulation of cellular metabolic process, cellular amino acid metabolic process, microtubule-based process, cell cycle process, histone modification, glutathione metabolism, and ABC transporters (Fig. 2, Additional file 8: Table S5 and Additional file 9: Table S6). These results were consistent with previous reports, which showed that HRW-induced proteomic changes were classified into such categories as defence and response to stress, sulfur compound metabolic process, oxidation-reduction process, and metal ion homeostasis [44]. In addition to Cd stress, H_2_ plays a protective role in many environmental stresses and diseases by regulating oxidation-reduction pathways in plants, fungi and animals. For example, H_2_ was found to play a role in alleviating salt stress by manipulating ZAT10/12-mediated antioxidant defence in Arabidopsis [11]. H_2_ was also found to enhance alfalfa plant tolerance to paraquat-induced oxidative stress [5]. In fungi, H_2_ regulates ROS metabolism via glutathione peroxidase under acetic acid stress [45]. In addition, H_2_ is an antioxidant in many animal models; thus, the antioxidant role of H_2_ is a conservative process in living cells responding to stress [3, 4].

In plants coping with Cd, many metabolic and gene expression processes have been identified, especially the synthesis of glutathione, phytochelatins, metallothioneins, and enzymes involved in the stress response [20]. Among the categories identified in this study, we noticed that the oxidation-reduction process and glutathione metabolism were significantly enriched, and these two processes were suggested to be important events in the plant response to Cd stress [46]. Glutathione synthesis was enhanced under Cd stress because of the requirement in chelation with Cd by PCs; meanwhile, Cd stress can promote sulfur assimilation to meet the need for glutathione [47]. In this study, most transcripts in the sulfur metabolism pathways showed an increase after HRW treatment, especially the genes involved in sulfate absorption, transport, and sulfur assimilation (Fig. 3, Additional file 10: Table S7). Moreover, the transcripts of the genes involved in glutathione synthesis were clearly enhanced by HRW under Cd stress (Fig. 3). In contrast to the results of most Cd-upregulated genes, there were also downregulated genes after Cd stress, such as *hGS*, which might indicate cellular toxicity by Cd. Interestingly, Cd-induced downregulation of *hGS* was alleviated when HRW pretreatment was applied, especially in RT-qPCR data, and the RNA-Seq data showed similar trends (increased by 8.3% compared to Cd stress samples), although it still showed slight downregulation compared to control samples (Additional file 10: Table S7). Generally, as a result, (h)GSH content was increased after HRW pretreatment (Fig. 6d, e). Similarly, a previous report showed higher levels of GSH content in Cd-stressed *Brassica campestris* after HRW pretreatment [15]. In addition, the sulfur contents both aboveground and underground were increased by HRW, especially after 12 h of Cd stress. Long-term Cd stress exhibited toxicity and decreased sulfur uptake, but not in the sample with HRW pretreatment (Additional file 11: Figure S4). These findings suggested that sulfur metabolism might be a key pathway downstream of H_2_ signalling during the Cd stress response. Furthermore, the uptake and root-to-shoot transport of sulfate is enhanced after Cd treatment through the regulation of endocellular networks in Arabidopsis [26]. The urgent need for sulfur generally leads to Cd tolerance, with two different phenomena: lower Cd hyperaccumulation. The phloem can be an important route for long-distance source-to-sink transport of PC-Cd in *Brassica napus* [48]. Relative studies are exciting for the purpose of metal phytoextraction through transgenic plant engineering [24]. In contrast, the application of sulfur can decrease cadmium translocation from roots to shoots by enhancing PC synthesis and increase cadmium tolerance by promoting the capacity of the GSH-AsA cycle and sulfur assimilation in *Brassica chinensis* [25]. Thus, there might be a series of pathways and complex mechanisms underlying Cd-induced signalling in different plants.

In addition to chelation, the antioxidant capacity of glutathione is one of the mechanisms of Cd-induced detoxification. Because of the multiple roles of GSH, normal plants exhibit fast GSH depletion after exposure to excess metals, and the GSH pool must be recovered by upregulating the GSH biosynthesis pathway and recycling by using the reducing power NADPH [24, 49]. In both the RNA-Seq and RT-qPCR results, the transcripts of genes involved in GSH and NADPH reduction were enhanced by Cd, especially under HRW pretreatment (Fig. 3). Furthermore, subsequent experiments showed that antioxidant enzymes were regulated, and oxidative stress was reduced, by HRW (Fig. 5, Additional file 12: Figure S5). Thus, in addition to the biosynthesis pathway, redox cycling was also enhanced by H_2_. The strong effect from the low concentration and short H_2_ exposure is likely caused by the direct signalling or signal regulation role of H_2_ [6, 50], though there might be other aspects involved in the relationship between H_2_ and glutathione metabolism. In brief, these findings indicated that H_2_ might act as a signal that mediates the tolerance of alfalfa to Cd by enhancing sulfur assimilation and glutathione-related pathways in plants coping with Cd [23, 26].

### H_2_-enhanced glutathione metabolism contributes to Cd tolerance by Antioxidation, chelation, and segregation

By using the specific (h)GSH synthesis inhibitor BSO [40], as well as mutant lines, the role of (h)GSH in H_2_-induced Cd tolerance was demonstrated. The results showed that in alfalfa, glutathione depletion by BSO inhibited seedling growth, which also blocked the alleviation of Cd stress by HRW (Fig. 4a, b). This result suggested that H_2_-enhanced Cd tolerance was at least partially mediated by improving (h)GSH metabolism. Under Cd stress, H_2_-induced (h)GSH metabolism can both cope with the oxidative damage indirectly caused by Cd and avoid intracellular Cd by chelation and compartmentation, which are considered to be the main mechanisms of Cd tolerance [38]. The content of TBARS, which indicates lipid peroxidation, was alleviated by HRW. Consistently, we noticed that HRW treatment resulted in a decrease in TBARS content under BSO treatment (Fig. 4c). Furthermore, evidence from the *A. thaliana cad2–1* line, a glutathione-deficient cadmium-sensitive mutant, showed similar results (Additional file 12: Figure S5). Interestingly, other pathways, such as response to oxidative stress (GO:0006979), were identified after HRW pretreatment under Cd stress (Fig. 2, Additional file 8: Table S5 and Additional file 9: Table S6). It is well known that Cd-induced oxidative stress is the result of ROS overproduction and antioxidant system disruption [20]. In this paper, the activities of SOD, APX, and CAT in alfalfa seedling roots were markedly inhibited in the early 24 h of Cd treatment, in contrast to the results observed in the HRW plus Cd treatment groups (Fig. 5e-g). It was suggested that the inhibition of SOD, APX, and CAT activities by Cd might be due to Cd toxicity and that H_2_-induced glutathione metabolism might take part in the alleviation of Cd-induced oxidative stress. These results are in accordance with the histochemical staining of lipid peroxidation, cell membrane integrity, and ROS (Fig. 5a-c). Interestingly, a recent report showed that sulfate can induce Cd tolerance by modulating sulfur metabolism and the antioxidant system [51]. In brief, these results suggested that glutathione metabolism played an important role in H_2_-induced Cd tolerance, even though H_2_ may have induced other antioxidant systems.

Moreover, previous reports indicated that GSH was preferentially allocated for PC synthesis destined for Cd chelation during the early stages of root exposure to Cd and that this effect caused a decrease in GSH levels without the activation of alternative pathways to complement its role as an antioxidant [49, 52]. In this study, γ-glutamyl contents were enhanced by HRW under Cd stress, especially in hGSH and (h)PC2 treatments (Fig. 6). The increased sulfhydryl group contents induced by HRW might be due to the regulation of gene transcription (Fig. 3). Furthermore, the sharp increase in cysteine content and notably reduced GSH and PC content mediated by BSO treatment suggested the potent effect of BSO in inhibiting GSH synthesis [41, 42]. The HRW plus BSO treatment showed little difference in thiol contents compared to the BSO-alone treatment, and these results suggested the prominent role of (h)GSH in H_2_-induced Cd tolerance (Figs. 4, 5, and Additional file 12: Figure S5). However, we noticed that hPC2 exhibited a significant increase in HRW under BSO and Cd treatment, which indicated the possible different roles of GSH and hGSH in physiological processes [40]. Overall, HRW-induced Cd tolerance was associated with Cd chelation by thiols and (h)PCs, which used (h)GSH as a precursor.

The results showed decreased Cd concentrations in alfalfa seedling roots after HRW pretreatment, suggesting that there might be other mechanisms for decreasing Cd uptake after HRW treatment (Fig. 7a). Similarly, in Chinese cabbage, HRW treatment can reduce Cd uptake [53]. These results might be partly due to the regulation of the transporters, such as natural resistance-associated macrophage protein 1 (NRAMP1) and heavy metal ATPases (HMAs), by H_2_ treatment, which finally resulted in the Cd-tolerant genotype (Fig. 4, Additional file 12: Figure S5 [53];). Moreover, Cd entered the cytoplasm and then chelated to (h)PC-Cd compounds, which were conducive to Cd compartmentalization by the transporters located on the vacuole membrane. In *A. thaliana*, 15 ABC proteins are characterized as ABCC family members. The PC transporters AtABCC1, AtABCC2 and AtABCC3 were identified to give *Arabidopsis* plants tolerance to Cd by sequestering the complex into vacuoles [54, 55]. Furthermore, some ABC transporters, such as AtPDR8, which is located at the plasma membrane and acts as a Cd extrusion pump, can mediate Cd resistance [56]. In this study, four ABC transporter genes annotated in the *M. truncatula* genome were upregulated by Cd and HRW (Fig. 7b-f). These results suggested that H_2_ also mediated the regulation of Cd transport in alfalfa seedlings coping with Cd. Thus, HRW treatment could decrease toxic Cd levels in the root cell cytoplasm by segregation after enhancing the chelation of Cd by glutathione and (h)PCs, which contributed to the tolerance of alfalfa to Cd stress.

## Conclusions

HRW-regulated DEGs in alfalfa seedling roots under Cd stress were identified using RNA-Seq and RT-qPCR analyses. Bioinformatics analysis indicated that HRW functions in oxidation-reduction processes, sulfur metabolism, and metal transport pathways in plants coping with Cd. Furthermore, through pharmacological evidence and experiments employing with mutant lines, (h)GSH metabolism was verified to be the key mediator in HRW-induced Cd tolerance through the regulation of oxidation-reduction status, Cd chelation, and compartmentation. The results obtained in this study may help to elucidate the mechanism governing H_2_ signalling.

## Methods

### Plant materials, growth conditions and treatments

Alfalfa (*M. sativa L. victoria*) seeds were obtained from Clover Seed & Turf Co. (Beijing, China; http://www.bjclover.com). Seeds were sterilized with 5% NaClO for 10 min and rinsed extensively in distilled water. After germinating for 1 d at 25 °C in darkness, uniform seeds were selected, transferred to nutrient medium [quarter-strength Hoagland’s solution, which included 1.31 mM KNO_3_, 1.94 mM Ca(NO_3_)_2_, 0.51 mM MgSO_4_, 0.25 mM KH_2_PO_4_, 0.025 mM FeSO_4_, 0.025 mM Na_2_EDTA (disodium ethylenediaminetetraacetic acid), 11.5 μM H_3_BO_3_, 2.29 μM MnSO_4_, 1.35 μM ZnSO_4_, 2.244 μM CuSO_4_, and 0.39 μM Na_2_MoO_4_; pH was adjusted to 6.0], and placed in a plastic chamber in the illuminating incubator (14/10-h day/night photoperiod with a light intensity of 200 μmol·m^− 2^·s^− 1^, 25/23 °C day/night temperatures). Five-day-old seedlings were then incubated with or without 10% concentration of saturated hydrogen-rich water (HRW, pH 6.01–6.03, [27]), _L_-buthionine-(*S,R*)-sulfoximine (BSO, 500 μM), and reduced glutathione (GSH, 1 mM) alone or in combination for 12 h, followed by 12, 24, 72, or 120 h of incubation in 100 μM CdCl_2_ in quarter-strength Hoagland’s solution (pH 6.0).

*A. thaliana cad2–1* mutant seeds used in this work were obtained from the Arabidopsis Biological Resource Center (http://www.arabidopsis.org/abrc). Wild-type (WT, Col-0) and *cad2–1* seeds were disinfected with 75% ethanol for 2 min, further sterilized with 3% NaClO for 20 min and rinsed in sterile water 3 times for 2 min each. Then, the seeds were kept at 4 °C for 2 d, followed by growth in 1/2 Murashige and Skoog (MS, pH 5.8) medium in a growth chamber with 14/10 h (day/night) regimes at 22 °C for 5 d. Uniform seedlings were selected for different pretreatments for 12 h and then treated with or without cadmium (CdCl_2_ 50 μM) for 3 or 5 d.

HRW was prepared by bubbling purified hydrogen gas (99.99%, v/v) into quarter-strength Hoagland’s solution, as previously described [5]. In our experimental conditions, the H_2_ concentration in freshly prepared HRW analysed by gas chromatography was 0.22 mM, and this concentration was maintained at a relatively constant level at 25 °C for at least 12 h [14]. After 12, 24, 72, or 120 h of Cd treatments, seedling tissues were sampled immediately to use or flash-frozen in liquid nitrogen and stored at − 80 °C prior to use.

### Analysis of H_2_ production in alfalfa

The endogenous H_2_ in alfalfa seeding roots was determined by gas chromatography (GC) as described by Jin et al. [5].

### RNA isolation, cDNA library construction, and Illumina deep sequencing

For RNA extraction, 5-day-old seedlings were pretreated with or without 10% HRW for 12 h with or without another 12 h of Cd stress. After various treatments, the seedling roots were harvested. For each treatment group, one mixed sample from three replicated experiments was used for RNA extraction. Total RNA was extracted using cetyltrimethylammonium bromide (CTAB, Sigma-Aldrich, St. Louis, MO, USA) following the manufacturer’s protocol. RNA integrity was confirmed by 1% agarose TBE (Tris-Borate-EDTA) gel electrophoresis (120 V, 15 min). The samples for transcriptome analysis were prepared using a TruSeq RNA Sample Preparation Kit (Illumina, San Diego, USA) following the manufacturer’s recommendations. Briefly, 5 μg of RNA was purified using oligo (dT) magnetic beads. By using the fragmentation buffer, the mRNA was fragmented into short fragments (approximately 200 bp), and then first-strand cDNA was synthesized with random hexamer-primer (N6 primers, Illumina, San Diego, USA) using the mRNA fragments as templates. Illumina-supplied buffer and Second Strand Master Mix were added to synthesize the second strand at 16 °C for 1 h. The double-stranded cDNAs were purified with a QiaQuick PCR extraction kit (Qiagen) and eluted with EB buffer for end repair and poly (A) addition. Finally, sequencing adapters were ligated to the 5′ and 3′ ends of the fragments. The fragments were purified by 2% agarose Tris-acetate-EDTA gel electrophoresis and enriched by 15 cycles of PCR amplification to create a cDNA library. The cDNA library was sequenced on the Illumina sequencing platform (HiSeq™ 2500), and 51 bp single-end reads were generated.

### Reverse-transcription quantitative PCR (RT-qPCR) analysis

For RT-qPCR, total RNA from root samples was isolated using Trizol reagent (Invitrogen) according to the manufacturer’s instructions. Approximately 2 μg of DNA-free total RNA was used for first-strand cDNA synthesis with the PrimeScript™ 1st Strand cDNA Synthesis Kit (TaKaRa Bio Inc., Dalian, China). The cDNA was diluted (1:5) and then amplified using specific primers, whose characteristics were evaluated (Additional file 13: Table S8) [57]. The reaction mixture included the following: 10 μL of 2 × SG Fast qPCR Master Mix (BBI Life Sciences Corporation), 0.8 μL of primer mix (10 μM), 1 μL of cDNA, and 8.2 μL of H_2_O. qPCR reactions were performed with a Mastercycler® EP Realplex real-time PCR system (Eppendorf, Hamburg, Germany). All experiments were performed with three independent biological replicates and three technical replicates. Data normalization was performed by using the statistical program geNorm, with two reference genes *Actin2* and *MSC27* [27, 58]. The expression levels of the corresponding genes are presented as values relative to the corresponding control samples (Con) under the indicated conditions.

### Bioinformatic analysis, differential expression, cluster analysis, and enrichment analysis

#### Quality control

Raw data (raw reads) in the fastq format were first processed using the NGS QC Toolkit [59]. In this step, clean data (clean reads) were obtained by removing reads containing adapters and ploy-N and with low quality from the raw data. All downstream analyses used clean data with high quality.

#### Mapping

Since the the great advantage of available *M. tructula* gene information and its close genetic relationship to *M. sativa*, sequencing reads were mapped to the *M. truncatula* (ftp://ftp.jcvi.org/pub/data/m_truncatula/Mt4.0/) genes and genome using bowtie2 [60] and Tophat (http://tophat.cbcb.umd.edu/) separately with default parameters that were slightly modified. The FPKM (fragments per kilobase of transcript, per million fragments mapped) and count value were calculated using eXpress [61]. Differential expression analysis was performed using the DEGseq R package. The *P* value was adjusted, and *P* value< 0.05 was set as the threshold for significantly differential expression. Hierarchical cluster analysis was used to identify differentially expressed genes with certain patterns of expression. Integrated Gene Ontology (GO) enrichment and Kyoto Encyclopedia of Genes and Genomes (KEGG) pathway analyses were employed to analyse the obtained differentially expressed genes (DEGs) by a muti-omics data analysis tool, OmicsBean (http://www.omicsbean.com:88/).

### Seedling growth, root elongation, and thiobarbituric acid-reactive substance (TBARS) analyses

After the different treatments, the root tissues of 30 seedlings were harvested and the fresh weight (FW) of aboveground and underground samples were determined. Root elongation was measured with ImageJ (available at http://rsb.info.nih.gov/ij) by taking the photos of seedlings after the 0 and 72 h of Cd treatment. Three replicated experiments were analysed (*n* > =12). Lipid peroxidation was estimated by measuring the amount of TBARS [62].

### Determination of glutathione by fluorescence microscopy

After the various treatments, Arabidopsis samples were loaded with 50 μM monochlorobimane in a phosphate buffer (pH 7.2) for 30 min, washed three times and analysed by microscopy (Axio Imager A1, Carl Zeiss, Germany; excitation 365 nm). The results were from three representative experiments (*n* = 12; [63]).

### Confocal determination of cadmium in plants

Cadmium concentration in Arabidopsis roots was detected by using a TCS-SP2 confocal laser scanning microscope (Leica Lasertechnik GmbH, Heidelberg, Germany). Samples were incubated in saline solution containing 0.04% (v/v) Leadmium™ Green (Invitrogen) for 30 min, washed three times and analysed (excitation 488 nm, emission 500–520 nm).

### Histochemical staining and enzymatic activities analyses

Histochemical detection of lipid peroxidation and loss of plasma membrane integrity in alfalfa and Arabidopsis seedling roots was performed with Schiff’s reagent and Evans blue, respectively, as previously described (*n* > 20) [5, 27]. Reactive oxygen species (ROS) production in alfalfa roots was detected by 3′3-diaminobenzidine tetrahydrochloride (DAB) staining [11, 27].

Antioxidant enzyme activities in alfalfa root samples were analysed according to previously reported methods. Guaiacol peroxidase (POD) and superoxide dismutase (SOD) activities were analysed by the methods described in previous reports [12, 14]. Ascorbate peroxidase (APX) and catalase (CAT) activities were measured as described previously [12]. Protein content was determined using Coomassie brilliant G250.

### Cysteine, γ-glutamylcysteine (γ-EC), (homo)glutathione ((h)GSH), and (homo)phytochelatin ((h)PC) analysis

Relative cysteine, *γ*-EC, (h)GSH, and (h)PC contents in alfalfa seedling roots were quantified using UPLC-ESI-QTOF/MS as described by Kühnlenz et al. [64], with minor modifications. Briefly, after different treatments, 200 mg of alfalfa seedling roots were harvested and ground to a fine powder in liquid nitrogen. The homogenous powder was extracted with 300 μL of 0.1% (v/v) trifluoroacetic acid containing 6.3 mM diethylenetriaminepentaacetic acid (DTPA) and 40 μM *N*-acetylcysteine (NAC) as the internal standard. After centrifugation at 4 °C and 13,000×*g* for 20 min, the supernatant was collected and reduced. Derivatization was performed as follows: 62.5 μL of the extract was added to 154 μL of 200 mM 4-(2-hydroxyethyl)-piperazine-1-propanesulfonic acid (EPPS) (6.3 mM DTPA, pH 8.2) and 6.25 μL of 20 mM Tris-(2-carbxyethyl)-phosphine (TCEP; in 200 mM EPPS, pH 8.2) and incubated at 45 °C for 10 min. Then, 5 μL of 50 mM monobromobimane (MBBR) was added and incubated at 45 °C for 30 min, followed by adding 25 μL of 1 M methanesulfonic acid to stop the reaction. The separation of the MBBR-labelled thiols was performed using a UPLC system equipped with an Acquity UPLC® BEH C18 column (1.8 μm, 2.1 × 100 mm, Waters Corporation, Milford, MA) with an injection volume of 2 μL. The following linear gradient of water (A, acidified with 0.1% formic acid, v/v) and acetonitrile (B, acidified with 0.1% formic acid, v/v) was employed at a flow of 0.4 mL/min: 98% A, 2% B for 1 min; a linear gradient to 60% B at 10 min; gradient to 95% B at 12 min; flushing with 95% B for 2 min; a gradient back to the initial conditions in 0.5 min; and an additional re-equilibration for 3.5 min. The thiols were detected with a Q-TOF Premier mass spectrometer equipped with an ESI-source (Waters) operated in the V+ mode [64]. The data were measured from three independent experiments with the mixture from two replicates for each.

### Determination of sulfur contents in the culture solution and plants

The contents of sulfate in nutrient medium were measured by the turbidimetric methods [65]. Total sulfur in plants were also estimated by turbidimetric methods after digestion and oxidation. For the determination of total sulfur in alfalfa aboveground and underground parts, samples were dried at 80 °C for 3 d, then the dry weight (DW) was determined. 0.1 g sample were digested in the solution contaning concentrated nitric acid and 60% strength perchloric acid (85:15, v/v) for 45 min. For determination, a 5 mL of nutrient solution or plant digestion solution was transferred to 25 mL volumetric flask, followed by the adding of 2.5 mL gum acacia (0.25%) solution and 1.0 g BaCl_2_ (sieved through 40–60 mm mesh). After dilute with deionized water to 25 mL, the flask were thoroughly shaken till BaCl_2_ completely dissolved. Within 10 min after the turbidity development, values were recorded at 415 nm with an UV–vis spectrometer (SP-752, Shanghai Spectrum, Shanghai, China). A blank was run simultaneously after each set of determination. For the protein-bound sulfur measurement, 0.1 g of alfalfa seedling samples were ground with liquid nitrogen and extracted with methanol until the precipitate turned white. After washed twice by acetone, the precipitation were dried by freeze-drying treatment to constant weight (about 2 h). Dried protein samples were analysed by a CHNSO analyzer (Vario EL cube, Elementar Analysensysteme GmbH, Germany) [66].

### Determination of Cd content

After treatments, seedling roots were washed three times with an EDTA-Na_2_ solution and rinsed briefly in de-ionized water. Then, the root tissues were kept at 60 °C for 3 d. The oven-dried roots (approximately 0.08 g) were cut into smaller pieces, weighed DW, and then digested with HNO_3_ using a microwave digestion system (Milestone Ethos T, Italy). The content of Cd in alfalfa seedling roots was measured by an inductively coupled plasma-optical emission spectrometer (ICP-OES, Perkin Elmer Optima 2100DV) with cadmium standard solution (GSB 04–1721-2004, National Standard Material Center, Beijing, China).

### Data treatment and statistical analysis

Unless noted, values are the mean ± SE of three independent experiments with at least three replicates for each. Data analyses were performed by using SPSS 20.0 software (IBM, Chicago, USA). The normality distribution of the data was inspected by the Shapiro-Wilk test. The homogeneity of the variances was checked by Levene’s test, and data were log transformed if necessary. Differences among treatments were analysed by one-way ANOVA, and *P* < 0.05 according to Duncan’s multiple range test was considered significant. * indicates significant differences (*P* < 0.05) according to Student’s *t*-test.

## Supplementary information

**Additional file 1:** **Figure S1.** H_2_ production in alfalfa seedling roots after 12 h of 100 μM Cd stress. Values are the means ± SE of three independent experiments with at least two replicates for each. * indicated significant differences (*P* < 0.05) according to *t*-test.

**Additional file 2:** **Table S1.** Summary of read numbers based on the RNA-Seq data from alfalfa seedling roots under hydrogen-rich water (HRW) and/or cadmium (Cd) treatment.

**Additional file 3:** **Table S2.** List of differentially expressed genes in Con→Cd vs Con→Con groups.

**Additional file 4:** **Table S3.** List of differentially expressed genes in HRW → Cd vs Con→Con groups.

**Additional file 5:** **Table S4.** List of differentially expressed genes in HRW → Con vs Con→Con groups.

**Additional file 6:** **Figure S2.** RT-qPCR experiments validated the quality of RNA-Seq data. Five-day-old seedlings were pretreated with HRW for 12 h followed by another 12 h treatment with 100 μM CdCl_2_. The sample without chemicals was the control (Con). Expression levels of corresponding genes are presented relative to the control samples, with normalized against the expression of two internal reference genes in each sample. Values are the means ± SE of three independent experiments with at least three replicates for each. Bars with different letters indicated significant differences (*P* < 0.05) according to Duncan’s multiple range test. The hollow dot indicated the log_2_ fold change of corresponding treatment vs Con → Con detected by RNA-Seq.

**Additional file 7:** **Figure S3.** Bioinformatics analysis of identified differentially expressed genes (DEGs). Counts for each category represent the total associated terms in the database. Terms with *P*-value < 0.05 are statistically significant.

**Additional file 8:** **Table S5.** Biological Process analysis of the 1968 DEGs.

**Additional file 9:** **Table S6.** Kyoto Encyclopedia of Genes and Genomes (KEGG) analysis of the 1968 DEGs.

**Additional file 10:** **Table S7.** Transcripts of gene in Fig. 3 significantly detected by RNA-Seq and its validation by RT-qPCR.

**Additional file 11:** **Figure S4.** Sulfur concentration in medium and alfalfa seedlings.

**Additional file 12:** **Figure S5.** Repression of endogenous glutathione synthesis results to HRW-insensitive phenotype in *Arabidopsis thaliana*. Five-day-old seedlings treated with or without 50 μM Cd for 3 (c) or 5 (a, b, d, e and f) days, which have or not pretreated with HRW or 1 mM GSH plus HRW for 12 h. (a) seedling growth of WT and *cad2–1* mutant plants. (b) primary root growth of WT and *cad2–1* mutant plants, Bars with different letters indicated significant differences (*P* < 0.05) according to Duncan’s multiple range test. (c) monochlorobimane fluorescence shows endogenous glutathione contents in WT and *cad2–1* seedling roots. (d) Cd concentration indicated by Leadmium™ Green AM dye in WT and *cad2–1* seedling roots. e and (f) histochemical staining by Evans blue (e) and Schiff’s reagent (f) showed plasma membrane integrity (e) and lipid peroxidation (f) in WT and *cad2–1* plant roots. Bars = 1 cm (a), 1.5 mm (c and d), and 3 mm (e and f).

**Additional file 13:** **Table S8.** The sequence and characteristics of primers for RT-qPCR.

## Data Availability

The data sets supporting the results of the present study are induced within this article (and its additional files). The data of RNA-Seq reads were deposited in the National Center for Biotechnology Information (NCBI) database under accession number (SRP181743). Biosample accessions were as follows: SAMN10785770, SAMN10785771, SAMN10785772, and SAMN10785773.

## Abbreviations

ABC transporter: ATP-binding cassette transporter
APX: ascorbate peroxidase
AsA: ascorbate
BP: biological process
BSO: _L_-buthionine-(*S,R*)-sulfoximine
CAT: catalase
CC: cellular function
Cd: cadmium
cDNA: complementary DNA
DEG: differentially expression gene
EDTA: ethylenediaminetetraacetic acid
G6PDH: glucose-6-phosphate-1-dehydrogenase
GO: gene ontology
GR: glutathione reductase
GSH: glutathione
GST: glutathione *S*-transferase
H_2_: molecular hydrogen
H_2_O_2_: hydrogen peroxide
hGSH: homoglutathione
hPC: homophytochelatin
HRW: hydrogen-rich water
KEGG: Kyoto Encyclopedia of Genes and Genomes
MBBR: monobromobimane
MF: molecular function
NAC: *N*-acetylcysteine
NADPH: nicotinamide adenine dinucleotide phosphate
PC: phytochelatin
PCS: phytochelatins synthase
POD: guaiacol peroxidase
RNA-Seq: RNA sequencing
ROS: reactive oxygen species
RT-qPCR: reverse-transcription quantitative PCR
SOD: superoxide dismutase
TBARS: thiobarbituric acid-reactive substances
UPLC-ESI-QTOF/MS: ultra-performance liquid chromatography-electrospray ionization-quadrupole time-of-flight tandem mass spectrometry
*γ*-EC: *γ*-glutamylcysteine
