# Supplementary material for: Transcriptome analysis reveals insight into molecular hydrogen-induced cadmium tolerance in alfalfa: the prominent role of sulfur and (homo)glutathione metabolism
Source: BMC Plant Biol. 2020 Feb 4;20:58. doi: 10.1186/s12870-020-2272-2 (PMC7001311; doi:10.1186/s12870-020-2272-2)
Supplement: Supplementary file 1 — Additional file 1: Figure S1. H2 production in alfalfa seedling roots after 12 h of 100 μM Cd stress. Values are the means ± SE of three independent experiments with at least two replicates for each. * indicated significant differences (P < 0.05) according to t-test. [file 12870_2020_2272_MOESM1_ESM.doc]

**Supplemental Figure S1**

**Figure S1.** H_2_ production in alfalfa seedling roots after 12 h of 100 μM CdCl_2_ stress. Values are the means ± SE of three independent experiments with at least two replicates for each. * indicated significant differences (*P*<0.05) according to *t*-test.

**Materials and Methods**

The endogenous H_2_ in alfalfa seeding roots was determined by gas chromatography (GC) as described by Jin et al. (2013).

Jin, Q., Zhu, K., Cui, W., Xie, Y., Han, B., Shen, W. (2013) Hydrogen gas acts as a novel bioactive molecule in enhancing plant tolerance to paraquat-induced oxidative signaling system. *Plant Cell Environ.* 36: 956–969.
