# Supplementary material for: Transcriptome analysis reveals insight into molecular hydrogen-induced cadmium tolerance in alfalfa: the prominent role of sulfur and (homo)glutathione metabolism
Source: BMC Plant Biol. 2020 Feb 4;20:58. doi: 10.1186/s12870-020-2272-2 (PMC7001311; doi:10.1186/s12870-020-2272-2)
Supplement: Supplementary file 2 — Additional file 2: Table S1. Summary of read numbers based on the RNA-Seq data from alfalfa seedling roots under hydrogen-rich water (HRW) and/or cadmium (Cd) treatment. [file 12870_2020_2272_MOESM2_ESM.doc]

**Supplemental Table S1**

Summary of read numbers based on the RNA-Seq data from alfalfa seedling roots under hydrogen-rich water (HRW) and/or cadmium (Cd) treatment.

| Sample | Total  Reads | Total  mapped | Multiple  mapped | Unique  mapped | Non-Splice  reads | Splice  reads |
| --- | --- | --- | --- | --- | --- | --- |
| Sample1 | 15,417,580 | 8,554,129 | 728,339 | 7,825,790 | 6,233,835 | 1,591,955 |
| Sample2 | 13,084,494 | 6,945,095 | 859,434 | 6,085,661 | 5,039,835 | 1,045,826 |
| Sample3 | 14,258,328 | 7,603,207 | 864,365 | 6,738,842 | 5,599,503 | 1,139,339 |
| Sample4 | 17,962,722 | 9,786,731 | 804,103 | 8,982,628 | 7,202,106 | 1,780,522 |

Sample1: Con → Con, 12 h in 1/4 Hoagland’s solution then change to fresh 1/4 Hoagland’s solution for another 12 h; Sample2: Con → Cd, 12 h in 1/4 Hoagland’s solution then change to another 12 h of Cd treatment in 1/4 Hoagland’s solution; Sample3: HRW → Cd, with 12 h of HRW pretreatment and followed by 12 h of Cd treatment; Sample4: HRW → Con, with 12 h of HRW pretreatment then change to 1/4 Hoagland’s solution for another 12 h.
