# Supplementary material for: Transcriptome analysis reveals insight into molecular hydrogen-induced cadmium tolerance in alfalfa: the prominent role of sulfur and (homo)glutathione metabolism
Source: BMC Plant Biol. 2020 Feb 4;20:58. doi: 10.1186/s12870-020-2272-2 (PMC7001311; doi:10.1186/s12870-020-2272-2)
Supplement: Supplementary file 6 — Additional file 6: Figure S2. RT-qPCR experiments validated the quality of RNA-Seq data. Five-day-old seedlings were pretreated with HRW for 12 h followed by another 12 h treatment with 100 μM CdCl2. The sample without chemicals was the control (Con). Expression levels of corresponding genes are presented relative to the control samples, with normalized against the expression of two internal reference genes in each sample. Values are the means ± SE of three independent experiments with at least three replicates for each. Bars with different letters indicated significant differences (P < 0.05) according to Duncan’s multiple range test. The hollow dot indicated the log2 fold change of corresponding treatment vs Con → Con detected by RNA-Seq. [file 12870_2020_2272_MOESM6_ESM.doc]

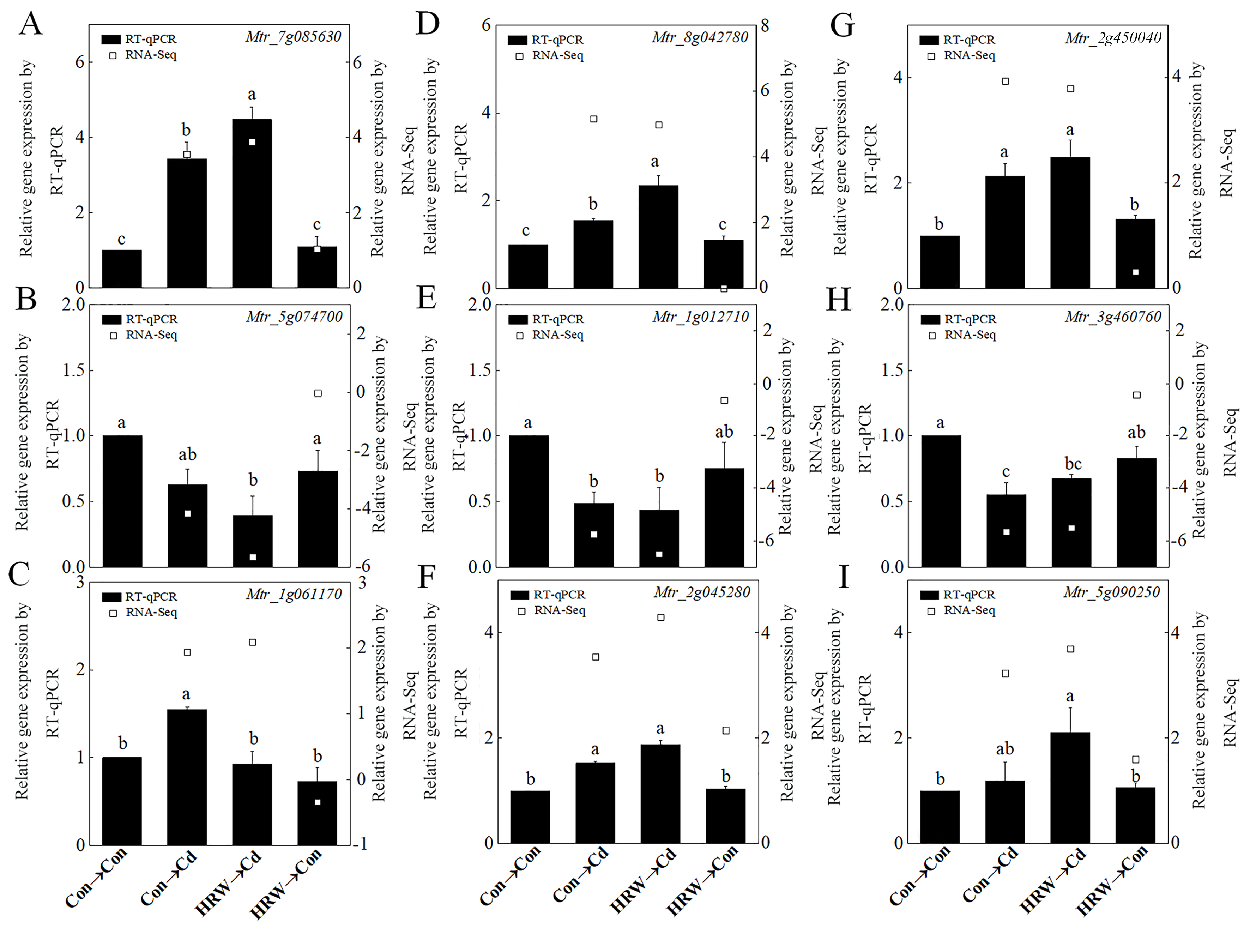
**Supplemental Figure S2**

**Figure S2.** RT-qPCR experiments validated the quality of RNA-Seq data. Five-day-old seedlings were pretreated with HRW for 12 h followed by another 12 h treatment with 100 μM CdCl_2_. The sample without chemicals was the control (Con). Expression levels of corresponding genes are presented relative to the control samples, with normalized against the expression of two internal reference genes in each sample. Values are the means ± SE of three independent experiments with at least three replicates for each. Bars with different letters indicated significant differences (*P*<0.05) according to Duncan’s multiple range test. The hollow dot indicated the log_2_ fold change of corresponding treatment vs Con → Con detected by RNA-Seq.
