# Supplementary material for: Transcriptome analysis reveals insight into molecular hydrogen-induced cadmium tolerance in alfalfa: the prominent role of sulfur and (homo)glutathione metabolism
Source: BMC Plant Biol. 2020 Feb 4;20:58. doi: 10.1186/s12870-020-2272-2 (PMC7001311; doi:10.1186/s12870-020-2272-2)
Supplement: Supplementary file 7 — Additional file 7: Figure S3. Bioinformatics analysis of identified differentially expressed genes (DEGs). Counts for each category represent the total associated terms in the database. Terms with P-value < 0.05 are statistically significant. [file 12870_2020_2272_MOESM7_ESM.doc]

**Supplemental Figure S3**

**
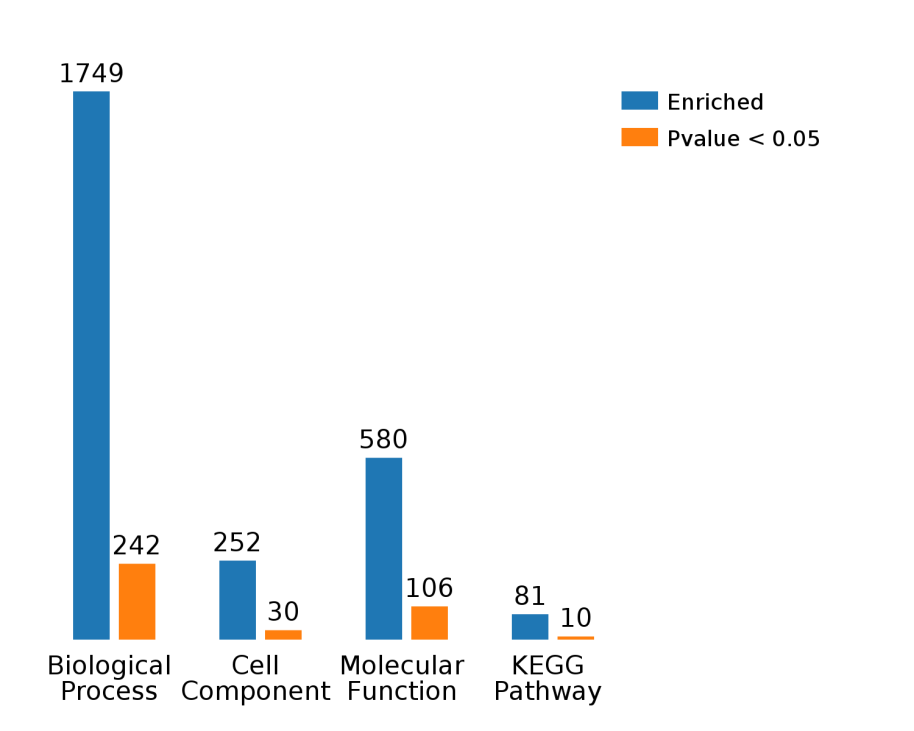
**

**Figure S3.** Bioinformatics analysis of identified differentially expressed genes (DEGs). Counts for each category represent the total associated terms in the database. Terms with P-value < 0.05 are statistically significant. The number on the top of each column indicated the counts of transcripts. KEGG, Kyoto Encyclopedia of Genes and Genomes.
