# Supplementary material for: Transcriptome analysis reveals insight into molecular hydrogen-induced cadmium tolerance in alfalfa: the prominent role of sulfur and (homo)glutathione metabolism
Source: BMC Plant Biol. 2020 Feb 4;20:58. doi: 10.1186/s12870-020-2272-2 (PMC7001311; doi:10.1186/s12870-020-2272-2)
Supplement: Supplementary file 11 — Additional file 11: Figure S4. Sulfur concentration in medium and alfalfa seedlings. [file 12870_2020_2272_MOESM11_ESM.doc]

**Supplemental Figure S4**

**
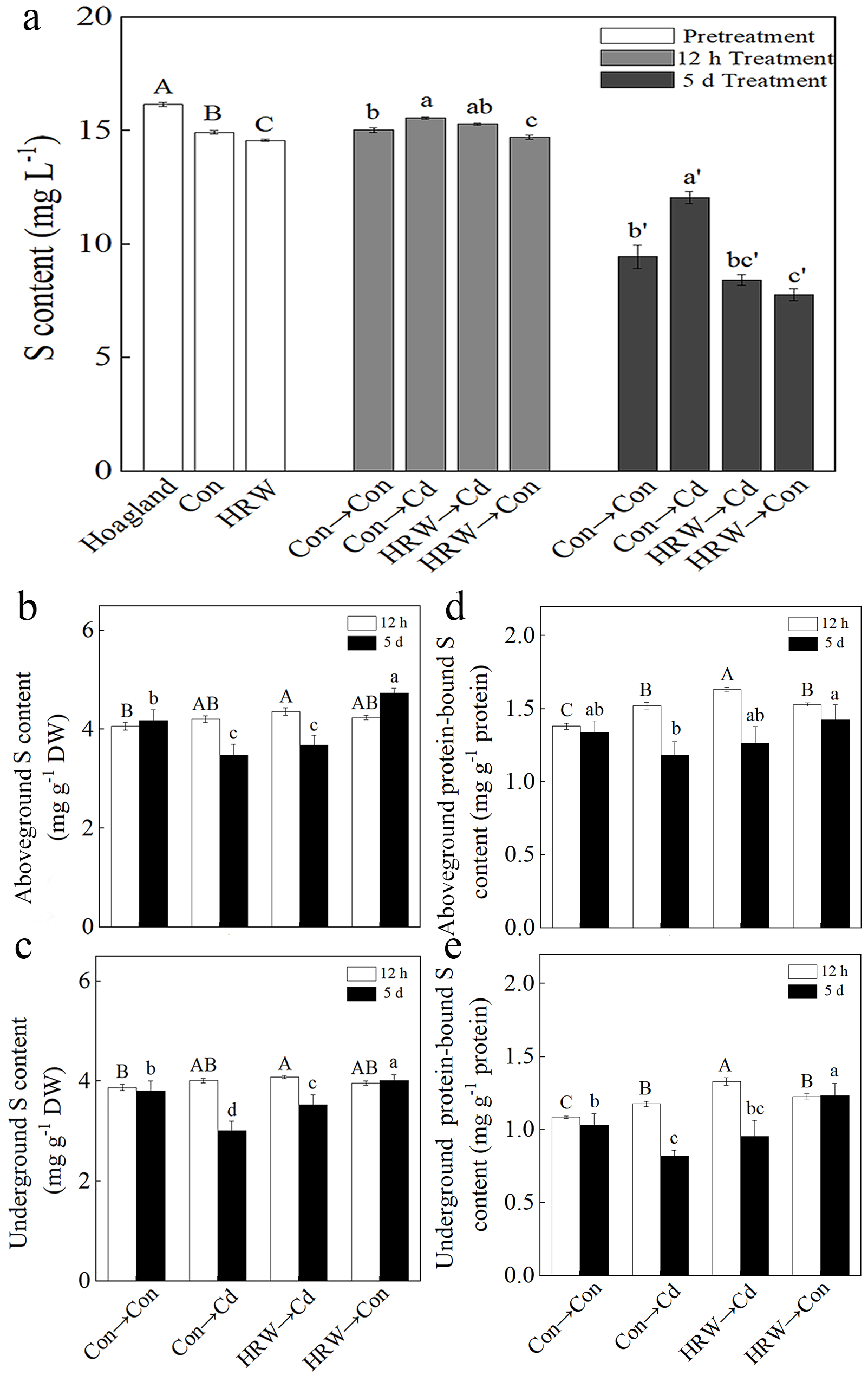
**

**Figure S4.** Sulfur concentration in medium and alfalfa seedlings. Five-day-old seedlings were pretreated with or without HRW for 12 h followed by another 12 h or 5 d treatment with or without 100 μM CdCl_2_. Values are the means ± SE of three independent experiments with at least three replicates for each. Bars with different letters indicated significant differences (*P*<0.05) according to Duncan’s multiple range test

**Determination of sulfur contents in the culture solution and plants**

The contents of sulfate in nutrient medium were measured by the turbidimetric methods (Per et al., 2017). Total sulfur in plants were also estimated by turbidimetric methods after digestion and oxidation. For the determination of total sulfur in alfalfa aboveground and underground parts, samples were dried at 80 °C for 3 d, then the dry weight (DW) was determined. 0.1 g sample were digested in the solution contaning concentrated nitric acid and 60% strength perchloric acid (85:15, v/v) for 45 min. For determination, a 5 mL of nutrient solution or plant digestion solution was transferred to 25 mL volumetric flask, followed by the adding of 2.5 mL gum acacia (0.25%) solution and 1.0 g BaCl_2_ (sieved through 40-60 mm mesh). After dilute with deionized water to 25 mL, the flask were thoroughly shaken till BaCl_2_ completely dissolved. Within 10 min after the turbidity development, values were recorded at 415 nm with an UV–vis spectrometer (SP-752, Shanghai Spectrum, Shanghai, China). A blank was run simultaneously after each set of determination. For the protein-bound sulfur measurement, 0.1 g of alfalfa seedling samples were ground with liquid nitrogen and extracted with methanol until the precipitate turned white. After washed twice by acetone, the precipitation were dried by freeze-drying treatment to constant weight (about 2 h). Dried protein samples were analyzed by a CHNSO analyzer (Vario EL cube, Elementar Analysensysteme GmbH, Germany) (Speiser et al., 2018).

Per TS, Masood A, Khan NA. Nitric oxide improves S-assimilation and GSH production to prevent inhibitory effects of cadmium stress on photosynthesis in mustard (*Brassica juncea* L.) Nitric Oxide. 2017;68:111–124.

Speiser A, Silbermann M, Dong Y, Haberland S, Uslu VV, Wang S, Bangash SAK, Reichelt M, Meyer AJ, Wirtz M, Hell R. Sulfur partitioning between glutathione and protein synthesis determines plant growth. Plant Physiol. 2018;177:927–37.
