# Supplementary material for: Transcriptome analysis reveals insight into molecular hydrogen-induced cadmium tolerance in alfalfa: the prominent role of sulfur and (homo)glutathione metabolism
Source: BMC Plant Biol. 2020 Feb 4;20:58. doi: 10.1186/s12870-020-2272-2 (PMC7001311; doi:10.1186/s12870-020-2272-2)
Supplement: Supplementary file 12 — Additional file 12: Figure S5. Repression of endogenous glutathione synthesis results to HRW-insensitive phenotype in Arabidopsis thaliana. Five-day-old seedlings treated with or without 50 μM Cd for 3 (c) or 5 (a, b, d, e and f) days, which have or not pretreated with HRW or 1 mM GSH plus HRW for 12 h. (a) seedling growth of WT and cad2–1 mutant plants. (b) primary root growth of WT and cad2–1 mutant plants, Bars with different letters indicated significant differences (P < 0.05) according to Duncan’s multiple range test. (c) monochlorobimane fluorescence shows endogenous glutathione contents in WT and cad2–1 seedling roots. (d) Cd concentration indicated by Leadmium™ Green AM dye in WT and cad2–1 seedling roots. e and (f) histochemical staining by Evans blue (e) and Schiff’s reagent (f) showed plasma membrane integrity (e) and lipid peroxidation (f) in WT and cad2–1 plant roots. Bars = 1 cm (a), 1.5 mm (c and d), and 3 mm (e and f). [file 12870_2020_2272_MOESM12_ESM.doc]

**Supplemental Figure S5**

**
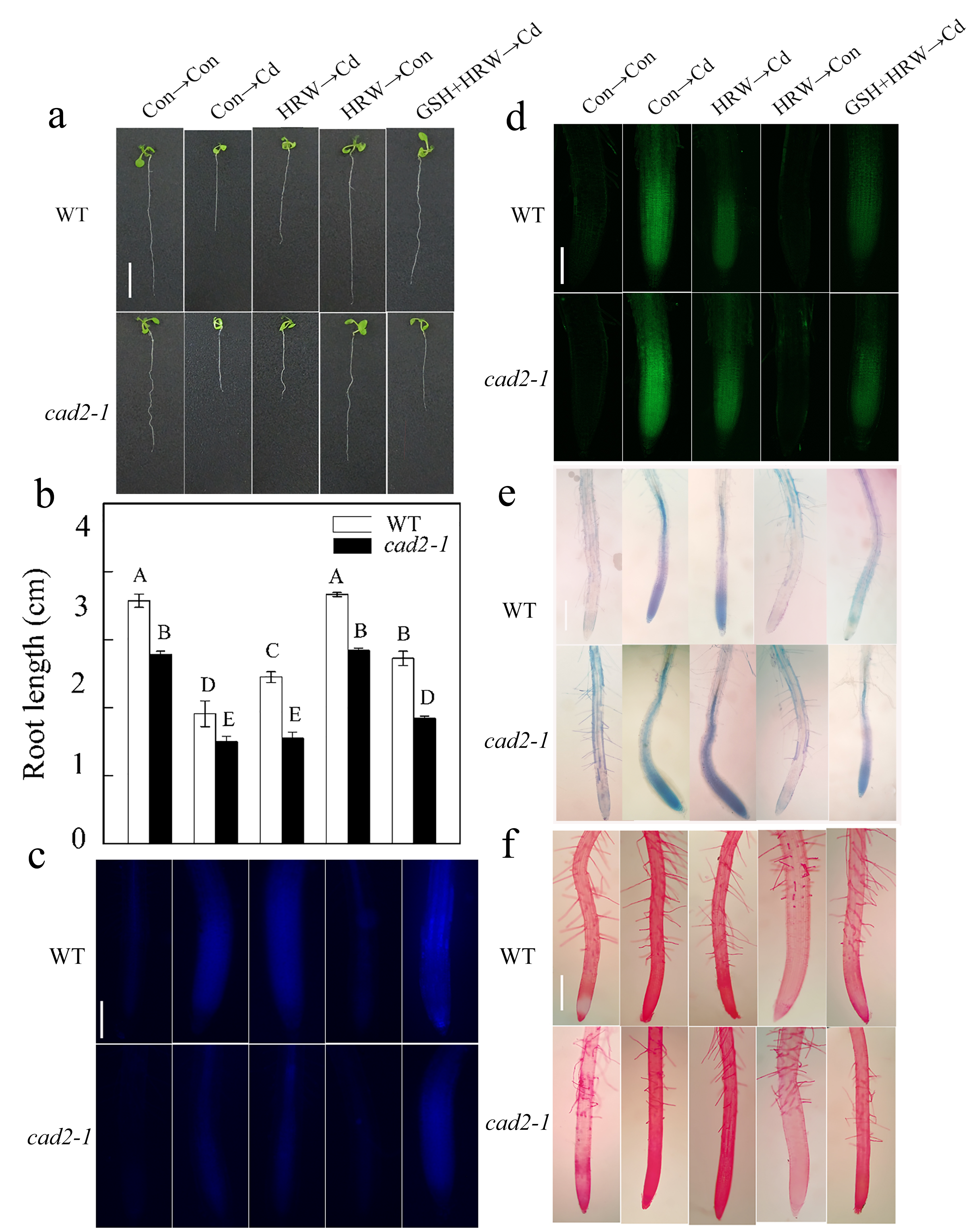
**

**Figure S5.** Repression of endogenous glutathione synthesis results to HRW-insensitive phenotype in *Arabidopsis thaliana*. Five-day-old seedlings treated with or without 50 μM Cd for 3 (**c**) or 5 (**a**, **b**, **d**, **e** and **f**) days, which have or not pretreated with HRW or 1 mM GSH plus HRW for 12 h. (**a**) seedling growth of WT and *cad2-1* mutant plants. (**b**) primary root growth of WT and *cad2-1* mutant plants, Bars with different letters indicated significant differences (*P*<0.05) according to Duncan’s multiple range test. (**c**) monochlorobimane fluorescence shows endogenous glutathione contents in WT and *cad2-1* seedling roots. (**d**) Cd accumulation indicated by Leadmium^TM^ Green AM dye in WT and *cad2-1* seedling roots. **e** and (**f**) histochemical staining by Evans blue (**e**) and Schiff’s reagent (**f**) showed plasma membrane integrity (**e**) and lipid peroxidation (**f**) in WT and *cad2-1* plant roots. Bars = 1 cm (**a**), 1.5 mm (**c** and **d**), and 3 mm (**e** and **f**).

**Materials and Methods**

**Plant Materials and Growth Conditions**

*Arabidopsis thaliana cad2-1* mutant seeds used in this work were obtained from the Arabidopsis Biological Resource Center (http://www.arabidopsis.org/abrc). Wild-type (WT, Col-0) and *cad2-1* seeds were disinfected with 75% ethanol for 2 min, further sterilized with 3% NaClO for 20 min and rinsed in sterile water for 3 times with 2 min for each. Then, kept at 4 °C for 2 d, followed by grown in 1/2 Murashige and Skoog (MS, pH5.8) medium in growth chamber with 14/10 h (day/night) regimes at 22 °C for 5 d. Uniform seedlings were selected for different pretreatment for 12 h, then treatment with or without cadmium (CdCl_2_ 50 µM) for 3 or 5 days.

**Determination of Glutathione by Fluorescence Microscopy**

After various treatments, samples were loaded with 50 μM monochlorobimane in a phosphate buffer (pH7.2) for 30 min, then washed three times and analyzed by microscopy (Axio imager A1, Carl Zeiss, Germany; excitation 365 nm). Results were from three representative experiments (n=12; Han et al., 2014).

**Confocal Determination of Cadmium Accumulation**

Cadmium accumulation in seedling roots was detected by using a TCS-SP2 confocal laser scanning microscope (Leica Lasertechnik GmbH, Heidelberg, Germany). Samples were incubation in saline solution containing 0.04% (v/v) Leadmium^TM^ Green (Invitrogen) for 30 min, then washed three times and analyzed (excitation 488 nm, emission 500-520 nm).

**Histochemical Analysis**

Histochemical detection of loss of plasma membrane integrity and lipid peroxidation was performed with Evans blue and Schiff’s reagent described by previous reports (Cui et al., 2013).

Cui W, Gao C, Fang P, Lin G, Shen W (2008) Alleviation of cadmium toxicity in *Medicago sativa* by hydrogen-rich water. J Hazard Mater 260: 715–724.

Han B, Yang Z, Xie Y, Nie L, Cui J, Shen W (2014) Arabidopsis HY1 confers cadmium tolerance by decreasing nitric oxide production and improving iron homeostasis. Mol Plant 7: 388–403.
