# Supplementary material for: Transcriptome analysis reveals insight into molecular hydrogen-induced cadmium tolerance in alfalfa: the prominent role of sulfur and (homo)glutathione metabolism
Source: BMC Plant Biol. 2020 Feb 4;20:58. doi: 10.1186/s12870-020-2272-2 (PMC7001311; doi:10.1186/s12870-020-2272-2)
Supplement: Supplementary file 13 — Additional file 13: Table S8. The sequence and characteristics of primers for RT-qPCR. [file 12870_2020_2272_MOESM13_ESM.doc]

**Supplemental Table S8**

The sequence and characteristics of primers for RT-qPCR.

| Primers | Gene ID or homolog gene | Sequence (5’→3’) | Amplicon length* / Product length** (bp) | Tm (°C) | Amplification efficiency (%) | r^2^ |
| --- | --- | --- | --- | --- | --- | --- |
| *high affinity sulfate transpoter type 1-F* | Mtr_3g073780 | TATTATCTCCGTGTTGAAGGC | 113 / 132 | 57.9 | 95.1 | 0.998 |
| *high affinity sulfate transpoter type 1-R* |  | CAATAAATTTGGCGACCAG |  | 54.9 |  |  |
| *ATP sulfurylase-F* | Mtr_1g102550 | CTCTTTCCCTTCTCACTCTCTC | 106 / 116 | 59.7 | 93.8 | 0.995 |
| *ATP sulfurylase-R* |  | AAGCCTCTGATTGGTTCTGG |  | 60.4 |  |  |
| *5’-adenylylsulfate reductase-F* | Mtr_2g023540 | TCGTATCGGATCTTCGTCT | 193 / 182 | 57.8 | 90.1 | 0.985 |
| *5’-adenylylsulfate reductase-R* |  | CTCAATAATCTCAGGTGCAAC |  | 56.9 |  |  |
| *sulfite reductase [ferredoxin] protein-F* | Mtr_4g077190 | AGTGCTCATCAGTTAGCCAT | 163 / 163 | 58.4 | 91.2 | 0.904 |
| *sulfite reductase [ferredoxin] protein-R* |  | CCTCAGTAACAGCACCGAGA |  | 60.7 |  |  |
| *O-acetylserine(thiol)lyase-F* | Mtr_5g006340 | CGCATTTGTTTCTGGGATAGG | 117 / 120 | 54.6 | 93.4 | 0.950 |
| *O-acetylserine(thiol)lyase-R* |  | AGCACTGGACTCTCTACTGG |  | 55.1 |  |  |
| *glutamate-cysteine ligase B-F* | Mtr_8g098350 | GACGCAACACCGCATAAC | 127 / 144 | 58.5 | 90.2 | 0.923 |
| *glutamate-cysteine ligase B-R* |  | CAACGATGAATCTACGACTACC |  | 57.5 |  |  |
| *glutathione synthetase-F* | Mtr_7g113890 | CTCTTGTCACTCAACTTCATAGG | 90 / 105 | 57.8 | 91.8 | 0.914 |
| *glutathione synthetase-R* |  | CAGCATTATTGGCAGGAACC |  | 58.6 |  |  |
| *phytochelatin synthase-F* | Mtr_7g097190 | TTGGGTTCCACTTACACTC | 179 / 171 | 56.1 | 110.2 | 0.954 |
| *phytochelatin synthase-R* |  | AAGGGACATCATCCATTAG |  | 53.0 |  |  |
| *homoglutathione synthetase-F* | Mtr_7g113880 | TTCTGGTGTTGGTGTTGTTATG | 105 / 138 | 58.5 | 90.2 | 0.985 |
| *homoglutathione synthetase-R* |  | TCGGCAGTATTGACAGGAAC |  | 59.2 |  |  |
| *glutathione S-transferase-F* | Mtr_2g070070 | ATATTATTGTGTCATGTGCTT | 188 / 168 | 52.7 | 107.3 | 0.935 |
| *glutathione S-transferase-R* |  | TAAAGAAAACAGTTAACAGCC |  | 53.6 |  |  |
| *glutathione reductase-F* | Mtr_6g033515 | TAACCAACCGAATGAATCTTACC | 159 / 176 | 57.9 | 92.3 | 0.992 |
| *glutathione reductase-R* |  | CCTCCTCACTTAGACCAACTAC |  | 58.7 |  |  |
| *NADP-dependent isocitrate dehydrogenase-F* | Mtr_2g062840 | ACTGGTGCTGGAGGTGTAG | 188 / 169 | 60.6 | 91.0 | 0.902 |
| *NADP-dependent isocitrate dehydrogenase-R* |  | CATTTGCTGTCATAAACTTCTTGG |  | 58.1 |  |  |
| *decarboxylating-like 6-phosphogluconate dehydrogenase-F* | Mtr_7g017900 | AACACGGAGAGGCGAGAG | 113 / 122 | 60.5 | 90.5 | 0.932 |
| *decarboxylating-like 6-phosphogluconate dehydrogenase-R* |  | CCACCAGGCATCATAGAAGG |  | 59.4 |  |  |
| *glucose-6-phosphate 1-dehydrogenase-F* | Mtr_7g111760 | TTCACTTTGTATTGCTGTCATAGG | 149 / 144 | 58.5 | 90.4 | 0.928 |
| *glucose-6-phosphate 1-dehydrogenase-R* |  | GGTCTTCGTCAGTTATATTCTTCC |  | 58.0 |  |  |
| *serine acetyltransferase-F* | Mtr_3g058410 | TTCTCGCAAACCAAACATTCC | 178 / 162 | 59.0 | 90.1 | 0.915 |
| *serine acetyltransferase-F* |  | CCACAAATCAACCTCAACACC |  | 59.0 |  |  |
| *cystathionine gamma-synthase-F* | Mtr_7g011230 | GGTTATGCACTCTTGTACGAA | 116 / 127 | 57.3 | 110.6 | 0.997 |
| *cystathionine gamma-synthase-R* |  | CGCCCAAAATATGATGCAA |  | 55.9 |  |  |
| *cystathionine beta-lyase-F* | Mtr_1g064320 | TTAGTATGCCGCTTTACCAG | 174 / 161 | 56.7 | 103.5 | 0.936 |
| *cystathionine beta-lyase-R* |  | TTAAAGCAGCCATTCCACT |  | 56.5 |  |  |
| *homocysteine S-methyltransferase-F* | Mtr_1g103290 | AAGTGTGGTGGGTATGGAATC | 109 / 101 | 59.4 | 93.8 | 0.925 |
| *homocysteine S-methyltransferase-R* |  | GGGAAGTAAAGAGGCATTTGG |  | 58.4 |  |  |
| *S-adenosylmethionine synthase-F* | Mtr_2g046710 | TTTCTCCGGTAAAGATCCAA | 186 / 175 | 55.7 | 101.2 | 0.991 |
| *S-adenosylmethionine synthase-R* |  | TTATCAGGGATCTTTCCGGTA |  | 57.4 |  |  |
| *nicotianamine synthase-F* | Mtr_1g084050 | TTTATTGGCTCAGGTCCAC | 153 / 132 | 56.5 | 90.2 | 0.923 |
| *nicotianamine synthase-R* |  | CATACGCTTTGATAATTCAGG |  | 53.9 |  |  |
| *ABC transporter (Mtr_1g086080)-F* | Mtr_1g086080 | TTTTCGGTTTCGTTCGGTT | 113 / 114 | 57.5 | 98.0 | 0.941 |
| *ABC transporter (Mtr_1g086080)-R* |  | AGGGAAGCAAGGCGTCTCA |  | 63.3 |  |  |
| *ABC transporter (Mtr_4g077930)-F* | Mtr_4g077930 | CAGTTTGGCTGTATGGTT | 133 / 124 | 54.2 | 99.1 | 0.923 |
| *ABC transporter (Mtr_4g077930)-R* |  | AGCACTTAGGCTTGGAGA |  | 57.1 |  |  |
| *ABC transporter (Mtr_4g124040)-F* | Mtr_4g124040 | ACATAGAGCTTAGGGAGG | 197 / 179 | 53.8 | 94.3 | 0.901 |
| *ABC transporter (Mtr_4g124040)-R* |  | GACACCGTCAATGAGAAC |  | 54.5 |  |  |
| *ABC transporter (Mtr_6g008800)-F* | Mtr_6g008800 | GACAAGCGTGGTTGTAGC | 194 / 158 | 57.8 | 108.2 | 0.914 |
| *ABC transporter (Mtr_6g008800)-R* |  | TTTCATGGCAAGAATCAGC |  | 55.5 |  |  |
| *ABC transporter (Mtr_6g008820)-F* | Mtr_4g008820 | TGTTGGGTCAGTGTTCGC | 156 / 141 | 59.6 | 96.5 | 0.991 |
| *ABC transporter (Mtr_6g008820)-R* |  | AACCCTTGAAAGATGATTGC |  | 55.7 |  |  |
| *Mtr_7g085630-F* | Mtr_7g085630 | GCTAATCACCCAACTGACC | 127 / 135 | 57.2 | 95.1 | 0.935 |
| *Mtr_7g085630-R* |  | GGATGTTCTGAACCCACT |  | 55.6 |  |  |
| *Mtr_5g074700-F* | Mtr_5g074700 | CGGCGACAATAATTTAGCG | 100 / 154 | 56.2 | 100.9 | 0.912 |
| *Mtr_5g074700-R* |  | CAACCTATCACTTTGAGAACCA |  | 57.5 |  |  |
| *Mtr_2g450040-F* | Mtr_2g450040 | TATGAGGATGATTGCTGGGAA | 117 / 114 | 58.3 | 90.2 | 0.902 |
| *Mtr_2g450040-R* |  | CCAACCACAAACAAACCTG |  | 56.5 |  |  |
| *Mtr_1g061170-F* | Mtr_1g061170 | GATCTTCTTCTGATGCAGGG | 113 / 124 | 57.1 | 95.4 | 0.965 |
| *Mtr_1g061170-R* |  | CATTCGTTGTTGTTGCTGTA |  | 56.1 |  |  |
| *Mtr_8g042780-F* | Mtr_8g042780 | CTCTATTCAAAGCCATTGGAC | 84 / 109 | 56.0 | 101.4 | 0.976 |
| *Mtr_8g042780-R* |  | CCGGTAGGAAAGAAGCATC |  | 56.9 |  |  |
| *Mtr_1g012710-F* | Mtr_1g012710 | CCTTTGTACTGCTATTAAGGCT | 107 / 114 | 57.3 | 90.3 | 0.914 |
| *Mtr_1g012710-R* |  | GGAAACCTGAAGGAACAGA |  | 56.1 |  |  |
| *Mtr_3g460760-F* | Mtr_3g460760 | CATTTCAGTTTCTCAAACGCTC | 118 / 110 | 57.5 | 93.8 | 0.986 |
| *Mtr_3g460760-R* |  | AAAGAATTGTAAGTGGTCGTG |  | 55.7 |  |  |
| *Mtr_2g045280-F* | Mtr_2g045280 | CACAAGCAAGCAATCAAGGATA | 103 / 109 | 58.7 | 104.8 | 0.974 |
| *Mtr_2g045280-R* |  | CAGCACCAAATGGACCTC |  | 57.4 |  |  |
| *Mtr_5g090250-F* | Mtr_5g090250 | AGGAAAGGTAGCAGAAGC | 125 / 124 | 55.7 | 90.2 | 0.985 |
| *Mtr_5g090250-R* |  | CTTTATCAACCCAAACCCTAAC |  | 56.3 |  |  |
| *MSC27-F* | X63872 | AGAATGGAATGTTGTGGGAGG | 113 / 122 | 59.7 | 93.9 | 0.932 |
| *MSC27-R* |  | GTCATCAACACCCTCATCTTCTC |  | 60.1 |  |  |
| *Actin2-F* | JQ028730 | AAAAGGATGCCTATGTTGGTG | 186 / 181 | 58.1 | 92.1 | 0.914 |
| *Actin2-R* |  | TAAGTGGAGCCTCAGTTAGAAGTA |  | 59.9 |  |  |

* Amplicon length based on *Medicago truncatula* gene (*MSC27* and *Actin2* are *Medicago sativa* genes)*.*

** PCR products on agarose gels determined using Quantity One software (Bio-Rad), with the samples from *Medicago sativa*.
